# From Impure to Purified Silver Nanoparticles: Advances and Timeline in Separation Methods

**DOI:** 10.3390/nano11123407

**Published:** 2021-12-16

**Authors:** Catarina S. M. Martins, Helena B. A. Sousa, João A. V. Prior

**Affiliations:** LAQV, REQUIMTE, Laboratory of Applied Chemistry, Department of Chemical Sciences, Faculty of Pharmacy of University of Porto, Rua de Jorge Viterbo Ferreira, n°. 228, 4050-313 Porto, Portugal; up201304479@edu.ff.up.pt (C.S.M.M.); hbffup@gmail.com (H.B.A.S.)

**Keywords:** silver nanoparticles, AgNPs, synthesis, separation, purification

## Abstract

AgNPs have exceptional characteristics that depend on their size and shape. Over the past years, there has been an exponential increase in applications of nanoparticles (NPs), especially the silver ones (AgNPs), in several areas, such as, for example, electronics; environmental, pharmaceutical, and toxicological applications; theragnostics; and medical treatments, among others. This growing use has led to a greater exposure of humans to AgNPs and a higher risk to human health and the environment. This risk becomes more aggravated when the AgNPs are used without purification or separation from the synthesis medium, in which the hazardous synthesis precursors remain unseparated from the NPs and constitute a severe risk for unnecessary environmental contamination. This review examines the situation of the available separation methods of AgNPs from crude suspensions or real samples. Different separation techniques are reviewed, and relevant data are discussed, with a focus on the sustainability and efficiency of AgNPs separation methods.

## 1. Introduction

In the literature, nanoparticles are defined as particles with a size between 1 and 100 nm and have been widely used because of their unique physical and chemical properties. Over the last years, their applications have been increasing in areas like medicine, the pharmaceutical industry, cosmetics, textiles, the food industry, and others of everyday products [1]. Nanoparticles (NPs) can be divided into two categories: the organics, and the inorganics. Belonging to the class of inorganics, noble metal NPs, especially silver NPs (AgNPs), are the most used [2], since these present intrinsic properties that make them exceptional. Apart from being useful as drug carriers or nanotheranostic sensors, some studies reported bactericidal, antifungal, and antiviral effects [3], which makes it possible to use them in the treatment of certain diseases and medical devices, such as wound and burn dressings, breathing masks, and implantable catheters [4].

AgNPs can be synthesized through two different strategies, the top-down and the bottom-up, with various materials and coatings, sizes, and shapes [5,6]. These strategies include a variety of physical, chemical, and biological methods. Recently, a sustainable method (a greener approach) to synthesize NPs emerged and enabled some reduction in the hazardous impact on the environment. This synthesis approach is based on green-chemistry concepts that rely on a “set of principles that reduces or eliminates the use or generation of hazardous substances in the design, manufacture and application of chemical products” [7]. This eco-friendly approach allows to synthesize NPs using solvents that are non-toxic to the environment, and using greener energy sources like microwave and ultrasound, at low-temperature and pressure conditions. Obviously, the green-based synthesis approaches that rely on relatively innocuous and bio-friendly reagents give raise to lower toxicity and higher biocompatibility of the AgNPs, due to the reagents’ chemical source and composition, determining the range of applications for the NPs. Additionally, by manipulating the synthesis parameters, it is possible to obtain AgNPs of different sizes and shapes [6]. This way, the controllable physicochemical properties can also be exploited for different applications of AgNPs.

Despite the reduced use of dangerous reagents or solvents in AgNPs synthesis through green-based synthesis [6], it may still be needed to use some reagents and precursors that have some toxicity, and, consequently, this might limit the possible applications of the nanomaterials in medical, biochemical, analytical, and clinical areas. For example, in bio-related applications, the by-products of the reactions’ synthesis might influence, interfere, or interact with natural occurring and complex processes in living cells or other organisms, originating unintentional effects, in comparison if only purified AgNPs were used. The common use of NaBH_4_ as a reducing agent in many syntheses might be a good example of a toxic reagent that, if not isolated from the raw suspension, will interfere with regular biological processes [6]. To circumvent this limitation, the purification of AgNPs from the crude synthesis media allows to obtain pure suspensions of the NPs, in opposition to using a mixture of the NPs and residues from the synthesis. The use of purified AgNPs in research studies increases by far the efficiency of the assays, allowing to reduce the number of assays necessary to conduct the research. Additionally, the purification of AgNPs allows to properly separate the synthesis’ remains, which are considered waste products, reducing their impact on the environment. Therefore, the search for novel separation techniques of AgNPs from the reaction medium is important, especially if it simultaneously allows for the sorting of NPs by size and shape, with this aspect being crucial for the proper usage of AgNPs in research studies.

Moreover, the growing use of AgNPs in consumer products leads to a higher release of these NPs to the environment, creating new hazards to health and the ecosystem, from surface waters to human consumption [8]. The persistent AgNPs exposure can be dangerous for humans because the accumulation of silver in the organism causes serious diseases, like argyria or argyrosis, which promote an irreversible discoloration of the skin or the eyes. The chemical monitoring of AgNPs in environmental, food, textiles, and biomedical samples is of paramount importance, and the analytical methods often rely on initial sample-treatment approaches based on separation or pre-concentration methods.

This review aimed at presenting a brief discussion of the principal methods used to separate AgNPs, between 2004 and 2020, and at providing an overview of the current developments in the separation techniques, pointing out advantages and disadvantages. Due to the wide range of distinct applications of AgNPs, this literature revision was restricted to the works where AgNPs, from crude suspensions or real samples, were used for the development, improvement, and optimization studies of several separation methods. The methods available for separation of AgNPs from the synthesis’ media are based on, for example, magnetic analytical schemes, hydrodynamic forces, chromatography, density gradient centrifugation, electrophoresis, selective precipitation, membrane filtration, and liquid extraction techniques, among others.

In 2015, a review about the use of AgNPs as nano-adsorbents for separation and preconcentration of environmental pollutants [9] highlighted the potential use of AgNPs to remove the contaminants of different environmental samples. In that review, the focus was on the use of AgNPs to separate and not its purification. Another work, by Wang et al. (2020), revised AgNPs and Ag ions speciation’ methods based exclusively on separation techniques coupled with atomic spectroscopy, but those only included solid-phase extraction, cation-exchange reactions, chromatography, and single-particle detection [10]. So, this revision work constitutes an advance in the general overview of eight different separation techniques, with a focus on the AgNPs separation from crude suspensions and real samples. For this purpose, scientific research was conducted at the global citation database Web of Science^TM^ using an advanced search for each separation method analyzed in this study. Considering the number of obtained articles, the analysis of each separation method was limited to some examples. A discussion about potentialities and limitations of the available methods was also included, while providing, at the same time, an overview of conceptual advances in a timeline approach.

## 2. Separation Methods of AgNPs

### 2.1. Magnetic-Based Schemes

Magnetic nanoparticles can be separated by magnetic forces according to their magnetic susceptibilities and/or their sizes [11]. Despite the fact that the theoretical discussion of the physical/magnetic details behind the separations is outside the scope of this review, very briefly, during capture of magnetic NPs there is a competition between magnetic forces and thermal diffusion [12]. The magnetic force (F_M_) acting on a nanoparticle can be calculated by the equation, F_M_ = µ_0_χV_p_ H.∇H, in which µ_0_ is the permeability of free space, χ is the magnetic susceptibility, V_p_ is the volume of the particle, and H is an external magnetic field [13,14]. More information can be consulted in the mentioned references, which explain the physical/magnetic details in a more-theoretical perspective. A revision on scientific literature about the separation of AgNPs by magnetic fields resulted in the compilation of the works presented in the Table 1, which is discussed more thoroughly in the following.

In 2013, Mwilu and his co-workers [15] developed a separation method using surface-modified magnetic-capture particles. The authors started by synthesizing unmodified magnetic particles (UMP), followed by glutathione-functionalized magnetic particles (GMP) and dopamine-functionalized magnetic particles (DMP). Next, they prepared a mixture of equal masses of DMP and GMP (Mix D–G) to assess their capabilities for separating AgNPs from aqueous media. For that purpose, the magnetic capturing particles were incubated with AgNPs suspensions (of different capping, namely, citrate and polyvinylpyrrolidone (PVP), and different sizes: 10 nm and 75 nm), and then the mixtures were exposed to a neodymium magnet, incorporated into a flow cell. After the application of a magnetic field, there was a visual clearing of the sample suspension, indicating the separation of the AgNPs from the medium flowing out the flow chamber. This methodology enabled separation of the eluate and the captured particles and quantification for total silver by inductively coupled plasma mass spectrometry (ICP-MS) upon acid digestion. The authors verified that the UMP-based magnetic particles allowed a high degree of selectivity for AgNPs over silver ions, with less than 5% of Ag^+^ adsorbed. The innovation of this work was the possibility of conducting pre-concentration of the separated AgNPs by magnetic particles, before quantification by ICP-MS, allowing the analysis of trace levels of AgNPs in samples, as well as the selective separation of nanosilver species in mixtures containing silver ions (Ag^+^). The selectivity and easiness of execution of the procedure was tested in the pre-concentration and detection of AgNPs content in environmental water samples, which revealed recoveries of spiked AgNPs > 96%. Additionally, the proposed methodology revealed precision and accuracy, free of matrix interference. One main drawback of the methodology was that AgNPs lose their individuality when they get adsorbed to the magnetic particles. After adsorption of AgNPs by the magnetic particles, further characterizations and use of the AgNPs are not possible due to the adsorption phenomena. Moreover, analysis of the particle-size distribution or nanoparticles’ quantification by particle counting using single-particle ICP-MS is also unfeasible. Single-particle inductively coupled plasma mass spectrometry (SP-ICP-MS) [17] has emerged as an instrumental method of analysis with proved potential [18,19] when it comes to a more-accurate approach for nanoparticle’s size characterization and quantification, which is of paramount importance in environmental and biological toxicological analyses. The SP-ICP-MS is a relatively recent analytical technique that provides information about the composition of each particle present in the sample, their size and size-distribution, their number density, and, also, their concentration.

The literature contains many works involving the separation of AgNPs by magnetic approaches but only when they are incorporated in magnetic nanocomposites, such as magnetic Fe_3_O_4_-Ag(0) [20]. Hence, the number of works involving the separation of single AgNPs by magnetic schemes is scarce, probably since AgNPs do have not magnetic properties.

### 2.2. Hydrodynamic Forces

Another method for the separation of AgNPs can be accomplished by exploiting the field-flow fractionation (FFF) technique, which is based on flow concepts [21]. FFF is a method of choice for the size-sorting of NPs based on their hydrodynamic size. The separation of NPs occurs inside a thin channel, where various fields actuate perpendicular to the laminar flow, causing different retention rates of the particles because of their distinct diffusion coefficients, accordingly with their size distribution and physicochemical properties [1,11]. This separation occurs because there is a balance between diffusibility and the external field forces, which leads to the formation of different equilibrium states among the laminar-flow streamlines. When compared with smaller NPs, the larger ones interact more strongly with the external field, being more retained in the accumulation wall, and hence they have longer elution times [11,21]. There are many other variants of FFF, including thermal FFF, sedimentation FFF (SdFFF), electrical FFF, magnetic FFF (MFFF), and flow FFF (F4) [21,22]. Additionally, F4 can be sub-divided into three technical variants: the asymmetrical F4 (AF4), the symmetrical F4, and the hollow fiber F4 (HF5) [23], which will be discussed together with selected literature works for better comprehension (Table 2).

In 2007, SdFFF was employed, by Kim et al. [24], to separate and determine the mean size and the size distributions of AgNPs of about 100 nm in diameter. SdFFF, also known as centrifugal FFF, separates nanoparticles according to their size and density. The separation occurs because there is a centrifugal force, which is generated inside the SdFFF channel when this is spinning at a high rate. The larger and denser nanoparticles are retained more inside the channel than the smallest nanoparticles, because the first ones accumulate on the wall. So, the smallest NPs are eluted first then the largest ones. This work aimed to implement the SdFFF method and optimize the experimental conditions for the AgNPs separation. Some experimental factors, among others, the flow rate, the carrier composition, and the field strength (channel rotation rate), were studied to find the optimal SdFFF conditions for separation of AgNPs. Regarding the proper carrier/eluent, which influences the NPs–NPs and NPs–channel wall interactions, the studies revealed that the separation of AgNPs was not achieved when using pure water as the carrier. Other aqueous-based compositions were tried out, with NaN_3_, SDS, or FL-70^TM^ commercial solution (containing a mixture of nonionic and anionic surfactants, including oleic acid, sodium carbonate, tergitol, tetrasodium EDTA, polyethylene glycol, and triethanolamine). However, only when using as carrier a 0.1% or 0.2% FL-70^TM^ commercial solution was the separation of two different populations of AgNPs achieved as intended, with more resolution when using a dilution of 0.1% for the FL-70 commercial solution. In this work, the authors also used mathematical-based deconvolution techniques to determine relative mass contents of AgNPs mixtures. Facing the obtained results, the authors recognized the potential of the methods but concluded that more work was needed for the optimization of experimental conditions, to make SdFFF a useful tool to separate and characterize metal NPs of a broader range of size and chemical nature.

**Figure 1:**
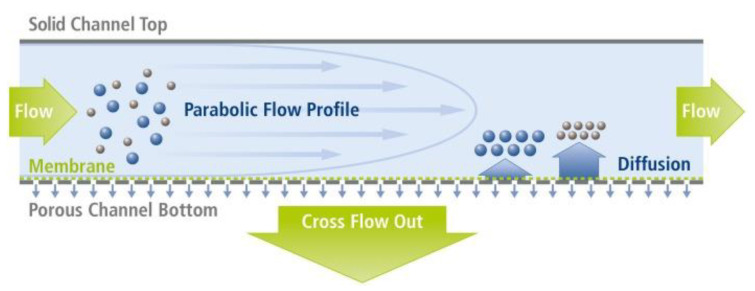
Scheme representative of asymmetric-flow field-flow fractionation. Reproduced from Ref. [30] with permission from Frontiers in Chemistry.

Considering FFF, the most widely used separation technique to isolate commercial NPs is AF4. Figure 1 represents the schematic principle of the separation by asymmetric-flow FFF.

This subtype of 4F bases its separation process on the force that is generated by a cross flow field inside the channel that concentrates the nanoparticles towards a membrane, which is located at the bottom wall of a chamber. This semi-permeable membrane prevents the NPs from passing through its pores but allows the exit of the solvent. Through the flow channel, the sample fractions are separated and eluted towards the detectors, out of the channel. The separation process can be resumed into three steps: the injection, the focusing, and the elution. In the literature, some studies reported the use of AF4 to separate AgNPs from food, nutraceuticals products, and beverages (Table 2). In fact, in 2011, for the first time, Bolea et al. [26] used AF4 coupled with ICP-MS to separate and quantify AgNPs in two consumer products, an antiseptic, and a dietary supplement. The parameters of the separation process that influenced the recovery and resolution were studied, comprising the mobile phase composition, the injection and focusing stages, and the membrane nature. The authors verified that to obtain reproducible results and high recoveries, the most-influencing factors were the mobile phase composition and the membrane nature. The optimal conditions obtained for the highest resolution in the separation of AgNPs were a mobile phase containing an anionic surfactant such as SDS at pH 8 and a polyether sulfone (PES) membrane. Recovery values of 83 ± 8% for the antiseptic and 93 ± 4% for the dietary supplement, with respect to the content of AgNPs, were achieved.

**Figure 2:**
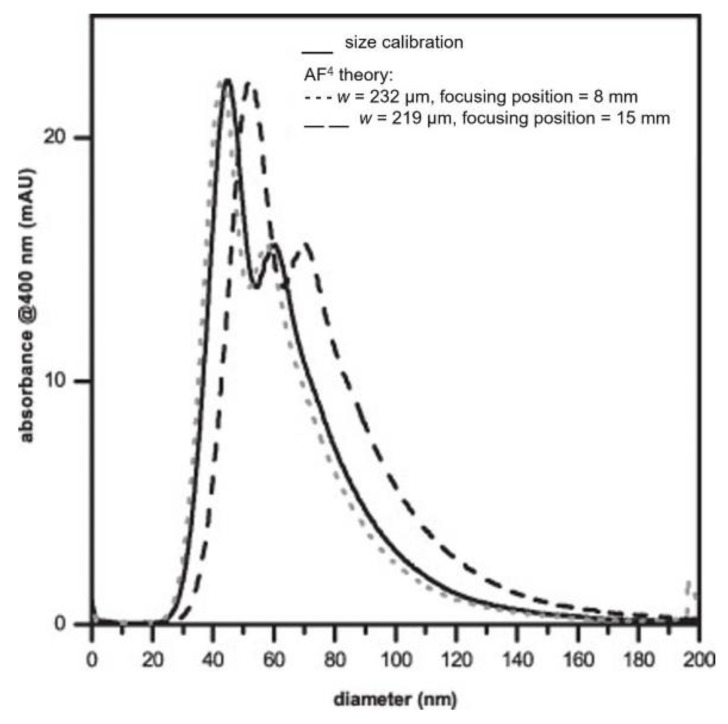
Size distributions of AgNPs by two different approaches: (i) size calibration using polystyrene beads (PSNPs) and (ii) conversion of retention times on AF4 analysis to diameters by AF4 theoretical calculations. Reprinted from [27] copyright (2013), with permission from Elsevier.

Two years later, in 2013, Loeschner et al. [27] developed and optimized an AF4-based method to separate AgNPs stabilized by PVP in aqueous medium. They studied the key factors that had an influence on the separation process, such as the carrier liquid composition, the type of membrane material, the cross flow rate, the spacer height, the focus flow rate, the focus time, and the injected mass. The optimized AF4 parameters, depicted in Table 2, originated relative recoveries > 95% approximately. After the optimization studies, the authors conducted four different approaches (i−iv), based on AF4, to determine the size distribution of AgNPs in suspensions. One of the methods (i) relied on the establishment of a calibration curve using different sizes of polystyrene (PSNP) beads as standards, namely, between 20−100 nm in diameter. After the confirmation that the same separation conditions of AgNPs could be used to properly separate the PSNP beads, a conversion of retention times to diameters was implemented to determine the size distribution of test samples (intensity of absorbance at 400 nm vs. diameter (nm)), assuming the same shape and water–surface interactions of AgNPs and PSNP beads. A second approach (ii) implied the conversion of retention times to hydrodynamic diameters, all dependent on AF4 theory, assuming the behavior of spherical particles at controlled conditions. However, as with most of the theoretical-based deductions, these are dependent on fixing some variables. This work evidenced the influence of the parameters’ focus position and channel height on the calculated size distributions. Considering that different nanomaterials for size-distribution calculations imply different separations conditions to achieve the closest to ideal, the previous-mentioned parameters must assume different values for calculations for each experimental separation. The results obtained using this approach were higher and lower than the results obtained by approach (i) when using some extreme values for focus position and channel height obtained by testing the AgNPs and PSNPs, respectively. Yet, overall, the results obtained by (i) and (ii) were similar (Figure 2).

**Figure 3:**
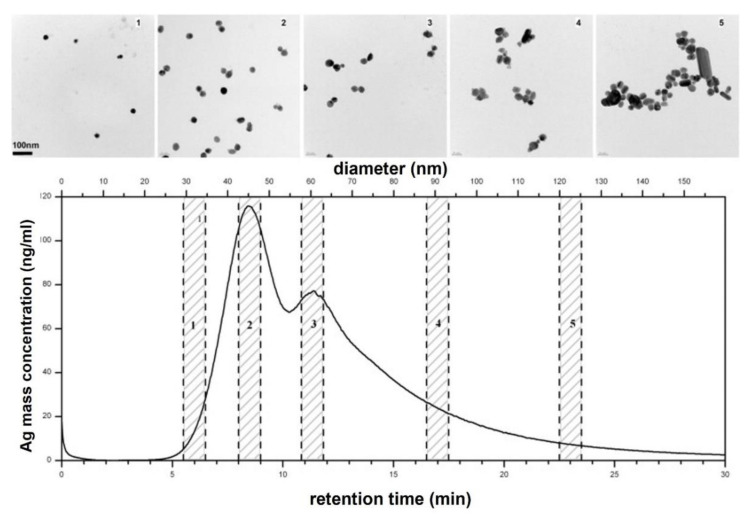
TEM images corresponding to collected fractions of AgNPs separated by AF4 at the conditions: m_inj_ = 10 µg, carrier liquid (NH_4_)_2_CO_3_ at pH 9, channel and cross flow rate 1.0 mL/min, spacer height 350 µm, PES membrane. Reprinted from [27] copyright (2013), with permission from Elsevier.

The third approach (iii) tested by the authors was based on independent measurements of the AgNPs sizes by TEM. For this purpose, the authors collected AF4-based separated fractions of AgNPs and analyzed each fraction by TEM. The results are depicted in Figure 3.

One important aspect about AF4 was that it could not differentiate larger particles from doublets or aggregates of AgNPs, which all appeared as larger diameters in the AF4 fractogram. However, by crossing the AF4 results with the optical spectrum, one could relate some signals apparently corresponding to higher-dimension particles to doublets or aggregates of more particles. The fourth approach (iv) tried by the authors involved in-line measurements with dynamic light scattering (DLS) or multi-angle light scattering (MALS), but these proved unable for the tested suspension of AgNPs. The DLS results revealed a lack of correlation between all ranges of available nanoparticles’ diameters, making impossible a complete size determination of a sample. On the other hand, with MALS, the scattered light intensity could not be correlated with the scattering angle due to plasmon resonance effects of AgNPs, for example. From this work, a very important conclusion was that all parameters influencing the AF4-based separation of AgNPs must be carefully studied and optimized or adapted whenever a new type of NPs is analyzed, due to different physical–chemical properties. Another limitation of the proposed method is related with obtaining the quantitative size information, that is, particle mass concentration-based size distributions. Some limitations of AF4 for the separation of AgNPs are due to electrostatic interactions between nanoparticles or nanoparticles and the membrane, caused by the surface charge of the AgNPs, which can hinder their separation behavior. Due to these possible interactions and expected different conditions for the ideal separation of AgNPs of different samples, the authors advised to use to approaches (i) and (ii) together with TEM imaging to successfully determine the size distribution of AgNPs suspensions.

In the next year, Ramos and co-workers [28] studied the feasibility of AF4 combined with ICP-MS for separation, characterization, and quantification of AgNPs in one beverage sample labelled as containing AgNPs ((i) Korean beer) and four commercial nutraceutical products ((ii) antibacterial, antiviral, and antifungal; (iii) antiseptic, disinfectant, and reinforced immune system; (iv) menstrual cycle regulation; and (v) flu prevention and allergies), which claimed to have biocide properties due to containing colloidal silver. In this work the authors emphasized the difficulty of separating, characterizing, and quantifying AgNPs in complex sample matrices like beverages and nutraceuticals, which can also contain other silver species and colloidal forms. In these cases, the sample treatments to extract the AgNPs without causing any kind of aggregation or oxidation processes present difficulties.

To determine the hydrodynamic size of the nanoparticles by AF4, the authors made use of the FFF equation and the Stokes–Einstein equation and compared with the values furnished by the manufacturer. The obtained results with this technique corroborated the TEM results, but with an advantage: the AF4 liquid samples could be analyzed directly, in opposition to what happens with TEM analysis in which the samples must be dried before imaging, with the risk for chemical changes in the sample in consequence of the drying process. After the optimization of the proposed method, the detection limit (LOD) obtained was <28 ng/L. The analytical recovery for total silver, for the nutraceutical product with “antiseptic, disinfectant, reinforce immune system” properties was 97 ± 2%, when spiked with 12.5 mg/L of ionic silver, and 106 ± 1%, with AgNPs of 40 nm of dimension. In the authors’ opinion, this methodology could become a significant alternative to more-conventional techniques, namely, ultracentrifugation and acid digestion, for silver speciation in complex matrix. The obtained results showed an efficient speciation of the AgNPs from other silver chemical forms, not being affected by the presence of ionic silver.

A miniaturized variant of the F4 technique also exists, which is named hollow-fiber flow field flow fractionation (HF5). In HF5, there is a hollow-fiber made of a porous membrane, where the separation occurs. A forced flow of a mobile phase through the fiber crosses the fiber towards the outlet with a laminar flow profile but also penetrates the pores of the tubular membrane, creating a radial flow (named cross-flow) that is perpendicular to the longitudinal carrier flow. In the process, the smaller nanoparticles (higher diffusion coefficient) of a sample reach faster the fiber outlet, whilst the higher-in-dimension or heavier nanoparticles take more time to complete elution. Thus, the size-fractionation of the nanoparticles is dependent on their diffusion coefficients accordingly with the radial cross-flow, having an influence on the molar mass and hydrodynamic radius of the nanoparticles, for example. In this technique, there are some improvements comparing with AF4, allowing for an increase in the separation efficiency in some cases: a low sample dilution due to the small channel volumes (≤100 µL) and low detector flow rates, allowing to couple to some mass-spectrometry detection methods; the hollow fibers are low-cost materials with a possible disposable usage [23]. The HF5 variant of the F4 technique was also exploited for the separation of AgNPs, with three reported studies presently found in the literature. The first one, described in 2015 from Marassi and co-workers [23], demonstrated the use of HF5 coupled to multi-angle light scattering (MALS) for size-separation and characterization of PVP-stabilized AgNPs in aqueous media. In this pioneering work, the results showed the presence of mainly two different average size populations of AgNPs: one with about 20 nm, and the other one with roughly 140 nm. The analytical recovery higher than 90% confirmed the efficiency of the proposed technique. This novel approach seems to be great to simultaneously separate by size and thoroughly characterize the AgNPs because it provides independent size information. This technique also adds the advantage of separating the Ag^+^ ions from AgNPs during the procedure, allowing to overcome potential hazards originated by Ag^+^ ions.

Additionally, in the same year, Saenmuangchin et al. [22] successfully developed a homemade HF5 coupled with ICP-MS for the separation of AgNPs, and it was able to circumvent the problem of different retention behaviors of AgNPs when different stabilizing agents were used in the synthesis of AgNPs. Firstly, the authors studied the influence of the carrier solution (FL-70 and TRIS buffer) and the stabilizing agent (tannic acid and citrate) on the retention behavior of AgNPs on the developed HF5 system. Depending on the carrier solution used, different elution profiles for the AgNPs were verified. When using the FL-70 carrier, the citrate- and tannic-acid-capped AgNPs were eluted from the separation system, being the separation depending on the nanoparticles size and on the nature of the capping. This constituted a problem, since AgNPs of similar sizes, but with different capping, had different retention times and were eluted in different fractions. When using the TRIS buffer solution, only the tannic-acid-stabilized AgNPs were eluted from the system. So, to circumvent this problem, tannic acid was added to the TRIS carrier solution to balance the retention behaviors of citrate- and tannic-acid-capped AgNPs. This approach had two purposes: (i) the citrate ligand exchange by tannic acid at the AgNPs surface and (ii) modification of the hollow fiber membrane to become negatively charged. The stabilizer’s exchange with tannic acid became necessary because citrate was known to have weak interactions with the nanoparticle’s surface, and, during the focusing phase in the HF5 separation procedure, citrate could be unbound from the surface of the nanoparticles, causing these to destabilize. Their destabilization could cause aggregation phenomena of the AgNPs and/or simultaneously increase the nanoparticle–membrane interaction, resulting in higher retention times. The strategy tested by the authors, with the use of 0.1 mM tannic acid in 30 mM TRIS buffer, resulted in similar retention behaviors and good recoveries for both tannic-acid and citrate-stabilized AgNPs. Yet, the proposed separation process must be optimized whenever other types of NPs with other surface coatings are involved, as already recognized by Loeschner et al. [27] when exploiting the AF4 separation-based concept. Overall, the work of Saenmuangchin et al. represents an interesting and valuable contribution for the separation of AgNPs based on the HF5 concept, by proposing a homemade HF5 system capable of separating citrate- and tannic-acid-capped AgNPs, reinforcing the potential of the FFF concept in the separation of nanoparticles, while at the same time providing a low-cost alternative to high-end FFF commercial systems.

**Figure 4:**
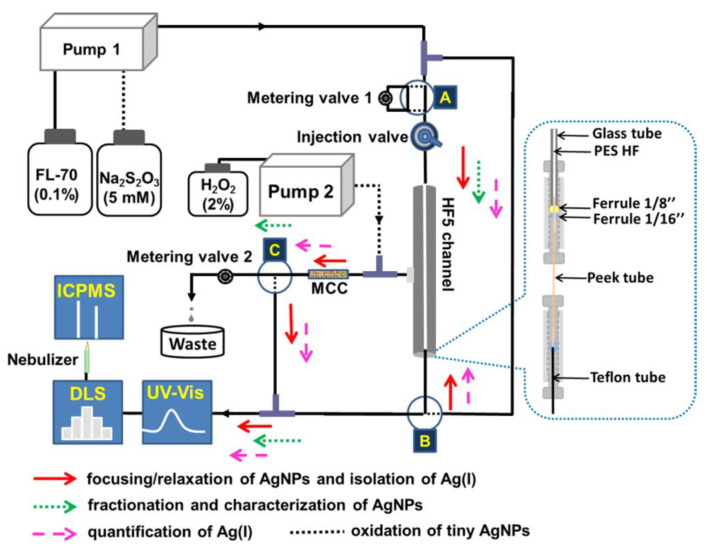
Schematic representation of the on-line coupled HF5/MCC-UV/DLS/ICPMS analytical system. Reprinted from [29] copyright (2015), with permission from ACS (further permission should be directed to the ACS).

Still in 2015, Tan et al. [29] employed, for the first time, the HF5 method for separation and fractionation of AgNPs (>2 nm) and various ionic silver species Ag(I), coupled with multiple detectors (namely, UV−Vis spectrometry, dynamic light scattering—DLS, and ICP-MS) for a full-spectrum speciation analysis and characterization of different sized AgNPs and Ag(I) species. To discriminate the sizes of AgNPs and Ag(I) species (<2 nm), a minicolumn packed with Amberlite IR120 resin was coupled to the HF5 system (Figure 4). The authors proceeded to the optimization of the HF5 system, and the optimal conditions obtained for the separation are described in Table 2. In the end, by resorting to multiple detectors, the separation, identification, quantification, and characterization of AgNPs and Ag(I) species were successfully achieved. By testing the developed analytical system with spiked lake and river water samples, the authors obtained recoveries between 70.7−108% for seven Ag species: five AgNPs (1.4 nm, 10 nm, 20 nm, 40 nm, and 60 nm), Ag(I), the adduct of Ag(I), and cysteine. The addition of a minicolumn packed with Amberlite IR120 resin to an on-line coupled HF5/MCC-UV/DLS/ICPMS analytical system for Ag(I) speciation analysis and characterization of AgNPs successfully enabled the multi-detection and characterization of different related silver species, including AgNPs of different sizes, reinforcing the potentialities of this novel NPs’ separation methodology.

Overall, the separation of AgNPs based on hydrodynamic forces provides researchers with good methods for that purpose, but the posterior application of the separated nanoparticles for other assays, in a purified format, was not verified in the reviewed works.

### 2.3. Chromatography

Chromatography is a separation technique based on the distribution of the components of a mixture between a fluid (mobile or eluent phase) and an adsorbent (stationary phase). The stationary phase can be a solid or liquid deposited in an inert solid, packed in a column or spread over a surface forming a thin layer [13]. Several scientific reports can be found in the literature about the use of different variants of chromatography to separate AgNPs from the crude suspension or other matrices (Table 3).

Soto-Alvaredo et al. (2013) [31] developed a method of coupling reversed-phase high-performance liquid chromatography (HPLC) with ICP-MS for the separation/detection of AgNPs and Ag(I) species. The direct coupling of HPLC to ICP-MS was accomplished by using only PEEK tubing connectors, a dual piston pump, and a six-way injection valve, between the column outlet and the nebulizer of ICP-MS. The studied AgNPs were stabilized with citrate and had sizes ranging between 10, 20, and 40 nm. In a single chromatographic run, all silver species (AgNPs and Ag(I)) were detected. For this purpose, thiosulfate was added into the mobile phase to elute Ag(I) species at the end of the separation, without influencing the stability of the AgNPs. The authors tested as a real sample sports’ socks, considering that these are examples of textiles known to contain AgNPs and other closely related additives. The proposed methodology allowed to obtain three different chromatogram peaks: the first two peaks were related to the presence of nanoparticles of different dimensions (the first peak in the range of 20−40 nm and the second one ~7 nm); the third peak was related to the presence of Ag(I) species. Interestingly, the authors could only conclude about the presence of silver species, as part of silver nanoparticles or bound to particles of other nature, as well the presence of silver ionic species. The origin of the detected Ag(I) species remained uncertain, as the AgNPs used in the study did not reveal to disintegrate and release silver ions, even during the extraction procedure. So, the authors believe that those species were already present in the textile sample and not formed during the extraction process. However, the assays carried out could not determine with certainty the origin of the ionic silver species. The results obtained from the extracts of sports socks showed that the quantitative data essentially depended on the extraction conditions, namely, the part of the textile sampled for extraction. So, despite the fact that more than 80% of recovery the of both the AgNPs and Ag(I) species was verified—and the calculated LOD was between 0.08 and 0.4 ng/L—it was proved that the extraction method had a marked influence in the developed methodology to be used as a routine laboratory assay in real samples.

Later, in 2016, C.A. Sötebier et al. [33] applied a combination of isotope dilution analysis (IDA) with the HPLC procedure reported by Soto-Alvaredo [31], mentioned above, and coupled to ICP-MS. The aim of the work was the separation and simultaneous quantification of AgNPs and Ag ions. The authors investigated the separation mechanism of AgNPs of different origins and dimensions, and Ag ions, through a comparative study of two different pore-size chromatographic columns: 1000 Å and 4000 Å. For the quantification of total Ag, AgNPs, and Ag ions concentrations, the authors resorted to a post-column IDA approach, using two different silver isotopes (^107^Ag/^109^Ag). The study showed a decrease in the recovery rates with the increase in AgNPs size when a 1000 Å column was used, whereas, with a 4000 Å column, the recovery rate could be considered independent of the AgNPs with sizes between 18 nm to 55 nm (<60 nm). The obtained results for a 1000 Å column could be due to interactions of the nanoparticles with the stationary phase, despite the fact that these often do not occur, considering that larger-sized nanoparticles can only interact with the larger pores of the column material. Regarding the LOD values, these were verified to increase with the particles’ sizes, when using the 1000 Å column, while, with the 4000 Å column, this effect only was verified for larger AgNPs. In fact, larger AgNPs were not eluted from the column of the 1000 Å column leading to conclude about the interaction more strongly with the smaller pores of the stationary phase, due to the higher negative charge and steric hindrance of larger AgNPs, which made it impossible to determine the exclusion limit for each of the columns. To around the problem of the detection of the exclusion value to the 1000 Å column, the authors performed HPLC-ICP-MS experiments in the single-particle mode (HPLC-spICP-MS) combination with IDA. They verified that a specific exclusion limit does not exist, which supports the argument that there are interactions of nanoparticles with the stationary phase. The authors pointed out that the proposed separation method could be applied to different nanoparticle systems like gold and polystyrene, but further investigation was required to eliminate the effects of interaction with the stationary phase, by choosing another eluent or column materials with even larger pore sizes.

**Figure 5:**
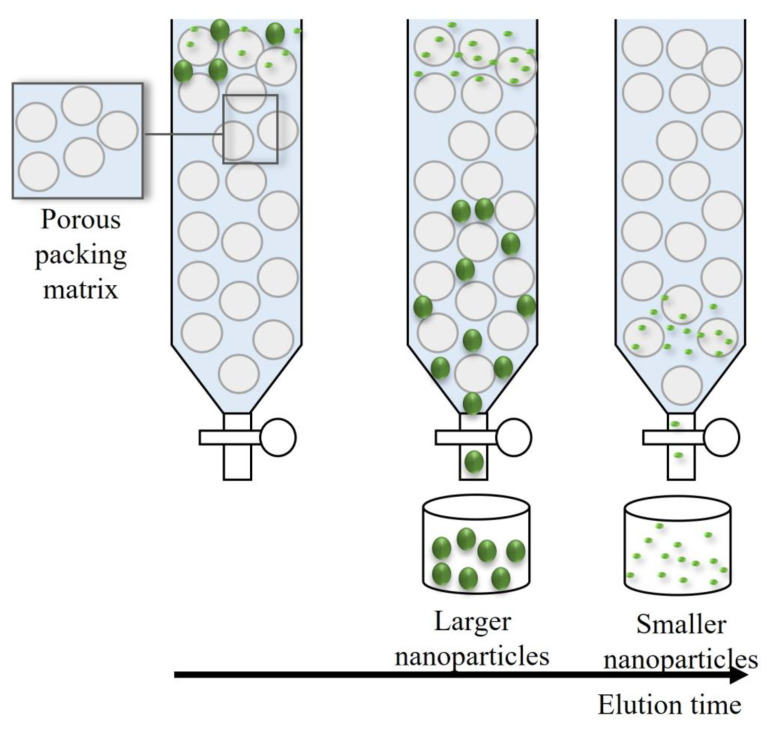
Representative scheme of SEC.

Among chromatographic-based techniques to fractionate NPs, size exclusion chromatography (SEC) is probably the most popular [13]. Over the years, this technique has been known by various names, namely, liquid-exclusion chromatography, gel-filtration chromatography, and gel-permeation chromatography [37]. In SEC, the separation is not based on the interaction of NPs with the stationary phase but on the differences in their hydrodynamic volumes [13]. The chromatography column is filled with a porous matrix, which creates flow channels. The smaller particles can permeate deep inside the column, because they have a minor diameter compared with the pore size of the matrix, whereas the larger ones are immediately excluded or conditioned to permeate between larger pores. The larger the particles, the shorter the retention time [1]. When compared with other separation methods, SEC presents several advantages such as being easy to scale-up, with a low chance of sample loss and less time for separation being needed. However, it may originate separations with low resolution, since it depends on certain factors such as the flow rate, the column dimensions, and the packing material, limiting to some extent its application [13,37]. Figure 5 shows a schematic representation of the separation principle of SEC.

In 2014, Zhou et al. [34] developed a novel SEC method for a rapid and high-resolution separation of dissoluble Ag(I) species from AgNPs, in five antibacterial products and three environmental water samples. The optimal conditions for obtaining the best results were found to be based on the use of a 500 Å pore-size amino column, an aqueous mobile phase containing 0.1% (*v/v*) FL-70^TM^, and 2 mM Na_2_S_2_O_3_, at a flow rate of 0.7 mL/min. As a result, AgNPs and Ag_2_S were eluted in one fraction, whereas dissoluble Ag(I) was eluted as a baseline separated peak. This efficient approach allowed to separate a whole range of AgNPs’ sizes between 1 to 100 nm, in only 5 min. The authors obtained excellent analytical recoveries, for environmental water samples, ranging from 84.7−96.4% for Ag(I) and 81.3−106.3% for nanoparticulate Ag. For the studied antibacterial products, the analytical recoveries obtained for Ag(I) ranged from 94.8−102.7%. For the first time, a successful separation between Ag(I) from ~1 nm Ag nanoclusters was reported. With these results the authors concluded that the proposed SEC-based methodology was a valuable alternative to use as a rapid and facile separation of dissoluble ions from the AgNPs bulk suspensions.

For the first time, in 2018, Dong et al. [35] developed a method by SEC coupled to ICP-MS, for accurate size characterization of AgNPs with biomolecule corona (AgNP@BCs) and for mass quantification of different Ag(I) species in biological tissues (rat and swine liver). The information provided by these analyses is of paramount importance for the understanding of AgNPs activity in vivo, that is, in which processes where the NPs are involved, interfere, or initiate in vivo, and which physical–chemical transformations they are subject to. The separation via SEC was based on the previous report of Zhou et al. [34]. The application of the proposed method to a rat liver after 24 h of exposure in vivo to the nanoparticles was verified as an excellent separation between Ag(I)-biomolecule complex and AgNP@BCs, which made possible the quantification of free Ag. The obtained concentrations were 36.4 ± 5.6 μg/g for the Ag(I)-biomolecule complex and 25.9 ± 2.8 μg/g for AgNP@BCs. To demonstrate the high accuracy of the proposed method to quantify the amount of Ag in vivo, the authors compared the total Ag mass from the two species (the Ag(I)-biomolecule complex and AgNP@BCs) derived from SEC-ICP-MS (62.2 ± 6.4 μg/g) with the total Ag content determined by microwave digestion followed by ICP-MS (64.3 ± 5.5 μg/g). Due to the similarity of the obtained values, it was possible to demonstrate the high precision of this method. This quantification allowed also to verify that after 24 h exposure to AgNPs, more than half (56.6%) of the total Ag accumulated in the liver was present as Ag(I).

Separation of AgNPs by size, through a counter-current chromatography (CCC) approach, a type of a support-free liquid chromatography, was studied in 2009 by Shen et al. [36]. This chromatography technique is composed by two immiscible solvents, which, after settling down, form two layers that act as the stationary phase and as the mobile phase. Usually, one layer is hydrophilic, while the other is hydrophobic. The separation of different chemical species or compounds by CCC occurs due to the different solubilities of the components between the stationary and the mobile phase. Because of the lack of a solid support to run the separation, there is no chance of irreversible adsorption of materials, which represents an advantage over other techniques [36,38]. The work of Shen et al. involved the synthesis of AgNPs modified with 11-mercaptoundecanoic acid (MUA), to study the potentialities of CCC in separating aqueous-dispersible nanoparticles. The solvent system was constituted by hexane/toluene 1:1, *v/v*, containing tetraoctylammonium bromide (TOAB) to act as a phase-transfer catalyst. The separation was achieved through ion-pair formation between tetraoctylammonium cations (TOA^+^) and the carboxyl group (anions) present on the AgNPs surfaces. To achieve the best separation and recovery conditions, several concentrations of TOAB were tested following a continuous extraction procedure. The assay revealed that the optimal concentration of TOAB for the continuous extraction was 0.02 mM. Following this, the authors also studied the influence of opting for continuous or stepwise extraction procedures, concluding that, after continuous extractions, a successful size discrimination was accomplished, with four fractions of AgNPs collected: 13.7 ± 1.9, 14.1 ± 3.5, 19.2 ± 4.3, and 22.2 ± 4.9 nm. On the other hand, after a stepwise extraction, the synthesized AgNPs sample only originated one fraction of 15.8 ± 5.3 nm. The obtained results demonstrated that the batch step-gradient extraction approach provided better size discrimination than the stepwise extraction. However, the application of the described separation methodology with real samples was not studied.

### 2.4. Density Gradient Centrifugation

Centrifugation is a technique used to separate particles from a solid–liquid mixture, according to their size, shape, and density. However, to achieve the separation of extreme small particles, like NPs, a centrifugal force is required to compensate or overcome the balance between gravitational forces, Brownian motions, and thermal diffusion, which allow the maintenance of the NPs in suspension [13]. The centrifugal forces originate sufficient energy to move the nanoparticles radially away from the axis of rotation, at pre-determined speeds, separating the NPs by shape and size. When the NPs have similar size and/or shape, the separation process by centrifugation is very difficult, and so a more-powerful technique like the density gradient centrifugation is required. This type of centrifugation is based on the creation of a density gradient [13]. The density gradient can be prepared, typically, with sucrose, glycerol, or another aqueous solution, and it is created inside of a centrifuge tube [1]. The solution that fills the centrifuge tube originates a decreasing density gradient from the bottom to the top of the tube [39].

Isopycnic centrifugation and rate zonal centrifugation are the two variants of density gradient centrifugation. In the isopycnic centrifugation, the process continues until most of the particles reach their isopycnic position in the centrifuge tube with the density gradient, that is, a position where their density equals the density of the medium. This type of centrifugation separates different particles based only on their different densities [39]. This way, one disadvantage of the isopycnic method is its incompatibility with metallic NPs separation, since these are denser than the highest densities attainable in aqueous media gradients (<1.7 g/cm^3^), making it not possible to separate metallic NPs with the isopycnic centrifugation method. For this purpose, rate zonal centrifugation is chosen [13]. Rate zonal centrifugation, also known as rate zonal ultracentrifugation, or typically as sucrose density gradient ultracentrifugation, is a classical separation and purification technique used in the laboratories to purify bulk suspensions, namely, nanoparticles [40]. In this technique the fractionation of nanoparticles occurs by size, shape, and density, sedimenting through the stationary gradient at different rates. This rate of sedimentation will depend on all the influencing factors, namely: the size, shape, and density of the nanoparticles; the viscosity and density of the gradient; and finally, the centrifugal force. For example, the larger nanoparticles will sediment closer to the bottom, and smaller ones will be retained closer to the top of the gradient [41]. Briefly, different solutions of sucrose concentrations (hence, densities) are prepared and layered on top of each other, from the bottom to the top of a centrifuge tube, originating a density gradient. Then, the sample containing AgNPs is dropped on top of the prepared sucrose gradient and centrifuged. Later, the separated layers are collected, washed repeatedly, and centrifuged again for further studies [42].

In general, centrifugation is considered a low-cost, straightforward, and appropriate method to isolate and purify AgNPs, especially to remove the residues from newly prepared suspensions [1,42]. Table 4 depicts the most-important aspects derived from the analysis of selected works in the literature where AgNPs were separated by centrifugation techniques. The selected works were reduced to the ones in which the authors included a detailed description of the experimental parameters used, to enable a proper analysis in this revision. Most of other works in the literature mentioning AgNPs separation by centrifugation do not include the valuable experimental data used for separation (parameters and conditions used in the centrifugations), making impossible its inclusion in the present review, which intends to be a comparison analysis.

A sucrose-density-gradient-centrifugation method was studied and optimized by Y. Asnaashari Kahnouji et al. [42] in 2019 for the separation of AgNPs produced through a chemical precipitation method and coated with chitosan, with sizes ranging between 15 and 235 nm. The best separation conditions were obtained with 10%, 20%, 30%, and 40% sucrose gradients, during 2 h at 6000 rpm and 5 °C. At the end of the separation process, the fractionated layers with the AgNPs were collected by syringe and washed thrice with deionized water. Then, the collect fractions were centrifuged to remove remaining residues, and, finally, they were dispersed in deionized water for characterization studies by FTIR, DLS, and UV–Vis analysis. The synthesized AgNPs characterized by DLS revealed a range of sizes between 15–235 nm, but most of the particles were in the range of 2.7–6.3 nm. Additionally, with the DLS analysis, the authors identified the size distribution of the separated AgNPs in the four sucrose layers. For the first layer, the nanoparticles ranged from 4.9 nm to 6.3 nm; in the second layer, the NPs were in the range of 3.9–4.9 nm; and, for the third layer, the nanoparticles were in the range of 2.7–3.4 nm. In the fourth layer, the NPs ranged from 98.3 to 235 nm. This study revealed that the AgNPs suffered from some type of capping disintegration, leading to the reduction in their hydrodynamic sizes after the separation. The authors related this observation with the washing step necessary after the separation, which lead to the loss of some of the chitosan stabilizing agent at the NPs surface.

In 2014, Lee and co-workers [43] synthesized Au and AgNPs of different sizes and shapes using *Magnolia kobus* leaf extract. To separate the Au and AgNPs synthesized from a plant-mediated process by size, the authors used sucrose density gradient centrifugation. The mother suspension of both Au and AgNPs was composed of NPs of different shapes such as pentagons, triangles, cubes, spheres, and hexagons of different sizes. A TEM analysis of the crude suspensions revealed nanoparticles sizes between 5 and 300 nm for AuNPs and 15 to 500 nm for the AgNPs. After some studies, the best separation results were obtained when using as conditions of the method, 40 min at 3500 rpm for AuNPs and 90 min at 3500 rpm for AgNPs. The smaller NPs were observed at the lower densities of the sucrose gradient, while the larger ones were observed at the higher-density layers. After the separation, for the AuNPs, the TEM analysis showed a particle size ranging from 52 to 117 nm and from 38 to 61 nm for AgNPs, from the lower- to the higher-density gradient. Up to date, all studies found in the literature have referred to the use of high values of centrifugal forces (5000–6000 rpm) for the successful separation of nanoparticles of different sizes. However, the described work allowed the use of 3500 rpm for the separation of nanoparticles synthesized using *Magnolia kobus*. Additionally, it was concluded in the work that the separation of the different shapes of the nanoparticles occurred in some way related to the density of the nanoparticles rather the shape; that is, there was the separation of NPs of different shapes along the different sucrose densities, but the analyzed TEM of the separated fractions revealed a mix of shapes per fraction, instead of a single shape type. For example, using a 30% sucrose concentration, it was possible to separate most of the small nanoparticles of spherical shape and, to a lesser extent, some triangle, and hexagon-shaped NPs.

Hyun et al. [44] proposed in 2015 the separation of AgNPs based on their surface-plasmon-resonance (SPR) bands. In the work, the authors synthesized AgNPs through a polyol reaction, with stabilization by PVP, to control the target SPR band of polydisperse AgNPs. The AgNPs obtained were quasi-sphere, and their size depended on the PVP concentration: when the PVP concentration decreased, the size of AgNPs increased. The quasi-sphere AgNPs can have a surface-plasmon-resonance band ranging from 320 to 450 nm. Afterwards, the synthesized raw AgNPs suspensions were subjected to separation by a centrifuging process. For that, the authors studied the influence of three different speed conditions (8000, 16,000, and 24,000 rpm) for separating AgNPs accordingly with the SPR bands. The monitoring of the SPR bands was conducted by UV–Vis spectrometry. The results revealed a successful separation based on SPR peaks. In fact, the AgNPs samples were separated accordingly with the SPR bands varying between 406 and 435 nm. Thus, this work positively revealed that the centrifugation-based separation technique was a promising approach to separate the AgNPs accordingly with the surface-plasmon-resonance band, and, thus, it allowed a more-thorough characterization of the optical properties of AgNPs.

The use of centrifugation-based methodologies to separate AgNPs allowed the recovery of the separated NPs fractions and, in some cases, the near preservation of their physical–chemical properties, enabling the posterior use of the purified AgNPs in specific chemical, biological, or biochemical assays. The discussed results place the centrifugation-based methodologies as one separation method that successfully separates NPs by size and shape, whilst efficient synthesis methodologies in order to obtain monodispersed, shape-segregated nanoparticle dispersions are still required to be improved.

### 2.5. Electrophoresis

Charged molecules or particles in a uniform electrical field can be separated by electrophoretic techniques. Among these techniques, gel electrophoresis is the most popular. In this technique, the charged particles are forced to migrate in a gel matrix, by an electric field, toward the electrode of the opposite polarity, being in the course separated into distinct bands depending on their charge, size, or shape [13,45]. The separations of NPs by gel electrophoresis with agarose gel or polyacrylamide gel are the most popular used, while tris-borate-EDTA (TBE) is the most common electrolyte [11,45]. In Table 5, one can find the collected information from the scientific literature regarding the separation of AgNPs by electrophoresis.

**Figure 6:**
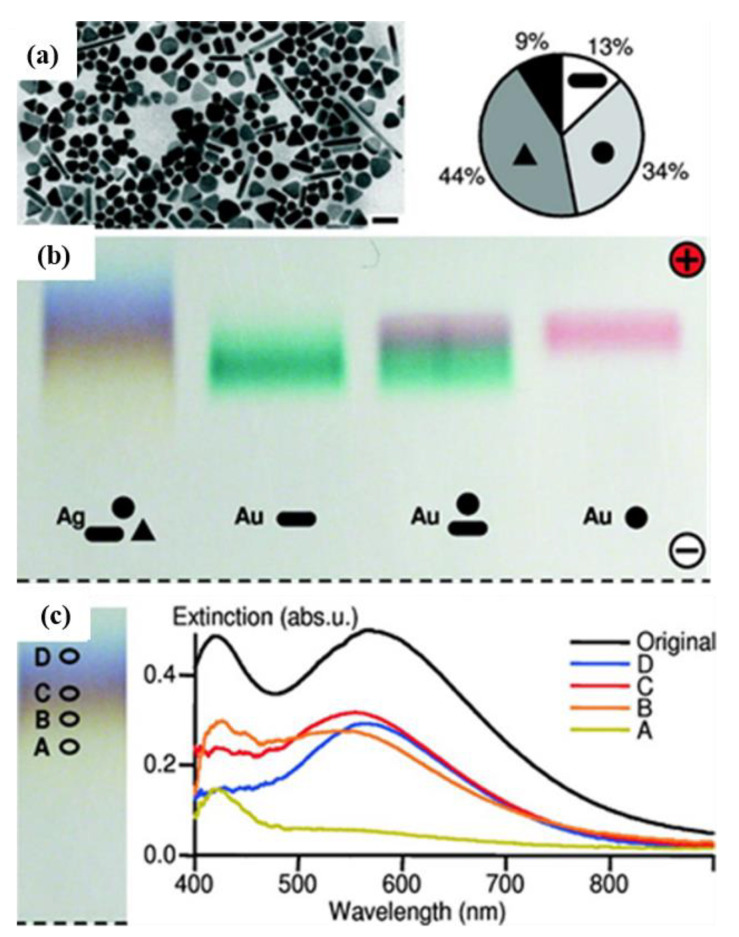
(**a**) TEM image of AgNPs sample and graphical distribution of the different shapes of the nanoparticles; (**b**) photograph of an agarose gel run for separation of nanoparticles (0.2% agarose, 30 min run, 150 V, 0.5× TBE buffer); (**c**) separated fractions of silver nanoparticles in agarose gel and their extinction spectra. Reprinted and adapted with permission from [46], copyright (2007) American Chemical Society.

The successful separation of AgNPs, coated with a charged polymer layer of polyethylene glycol (PEG), by agarose gel electrophoresis, according to their size and shape, was demonstrated by Hanauer et al. [46] in 2007. In fact, in this work, the authors studied the potentiality of electrophoretic-based separation for AuNPs and AgNPs of very different shapes and sizes. The TEM analysis of the sample before the separation revealed the presence of 13% rods, 34% spheres (including hexagons), 44% triangles, and 9% other shapes (Figure 6a,b). The separation results were monitored spectrophotometrically of the surface-plasmon-resonance bands (and recorded by photographs of the gel) and confirmed by TEM. With a true-color photograph of the gel, it was possible to verify that the authors achieved the separation of the NPs with a 0.2% agarose gel run, for 30 min at 150 V, in 0.5× TBE buffer (pH ≈ 9). The photos of the resultant gel showed four different colors, which were related to the AgNPs separation according to their morphology and size. The colors that appeared in the gel were due to the size- and shape-dependent optical properties of AgNPs, but it was not possible to obtain a distinct separation of these band colors (Figure 6c).

After the separation by gel electrophoresis, the TEM results revealed the presence of rods in the fraction with the particles with the lowest mobility, while there were spheres and triangles in the faster fractions. The average particle size was lower in the slowest fraction (41 ± 2 nm) and higher in the faster fraction (65 ± 2 nm). The authors demonstrated the capacity to separate AgNPs through a gel electrophoresis method, according with their size and shape.

Another potential technique to separate AgNPs is capillary electrophoresis (CE). In CE, the separation process is based on the difference in the electrophoretic mobility of the NPs [48,49], and thus separation occurs accordingly with size and/or surface charge density. In the literature, there are some examples of CE applications, such as the work of Liu and co-workers [47], in 2005, which combined CE with a diode-array detection (DAD) system, allowing to achieve simultaneously the separation and characterization of AgNPs. To obtain a fully resolved separation, the authors found that the addition of an anionic surfactant, like sodium dodecyl sulphate (SDS), in the running electrolyte, enhanced the resolution of the separation, because it prevented the coagulation of AgNPs during the separation process. The optimal concentration of SDS was found to be 20 mM. This work, without question, constituted an important landmark in the field, because it showed that the combination of CE with DAD system was a powerful method to simultaneous separate and characterize AgNPs.

In 2016, Qu and co-workers [48] combined CE with ICP-MS for a rapid separation and quantification of AgNPs and Ag ions, in consumer products (six dietary supplements). The AgNPs analyzed were of different capping nature and were coated with citric acid, lipoic acid, PVP, and bovine serum albumin (BSA). The nanoparticles coated with BSA are more likely to adsorb free ionic Ag^+^ on their surface, resulting in a significant underestimation of the amount of Ag^+^ and overestimation of the amount of AgNPs. To facilitate the separation, and to keep the oxidation state of silver, preventing interaction of BSA with Ag^+^, the compound tiopronin was added to the background electrolyte, in the concentration of 1 mM. Even with an excess amount of BSA, the authors obtained a recovery > 93%, for both ionic silver and AgNPs. With further studies in the commercially available products, they achieved the speciation of AgNPs and Ag^+^ in six minutes, under optimized conditions, detailed in Table 5. The robustness of the method was evaluated by the analysis of six dietary supplements by the proposed system CE-ICP-MS. The obtained results constituted by the amounts of nanoparticulate Ag and free Ag ions were compared with ICP-MS analysis of total Ag after the acid digestion of the same samples. A good accordance between the sum of the amounts of ionic and AgNPs by CE-ICP-MS and the obtained total silver quantities by ICP-MS after acid digestion was obtained.

More recently, in 2017, Fa et al. [49], with the aim of simultaneously obtaining information about zeta potential, size distribution, and the colloidal stability of AgNPs, used CE to separate AgNPs synthesized at pH 5.0 and 10.0 with honey or glucose as reducing agents (green-chemistry approach). In the separation, an electrolyte solution composed of 20 mM sodium borate and 20 mM SDS at pH 8.5 was used. The developed method allowed the separation of AgNPs within a short run time (<12 min). The obtained characterization results with the proposed method were compared with the DLS and TEM analysis. The electrophoretic mobility and zeta potential values were calculated through Smoluchowski’s equation and Ohshima’s equation, respectively. The calculated electrophoretic-mobility values were in accordance with the ones obtained by DLS. Additionally, Ohshima’s equation provided similar results for the zeta potential, when compared to those obtained by conventional characterization techniques. The proposed method required minimal volumes of samples and reagents, produced small amounts of residues, was easily handled and low-cost, constituting a good alternative for the separation of green-synthetized AgNPs while maintaining the same sustainability standards imposed by green chemistry.

One other variant of CE is micellar electrokinetic chromatography (MEKC). Nanoparticles are known to be positively or negatively charged, accordingly with the chemical nature of their capping or stabilizing agent used during the synthesis process. These capping agents promote the stabilization of the NPs via electrostatic or steric repulsion. The importance of the charge relies on preventing the NPs aggregation over the time and also gives stability to the nanoparticles while in suspension [6]. MEKC is a hybrid technique that combines an electrophoresis process with chromatography, and it can be used for the separation of AgNPs, because they are charged.

In MEKC, both ionic and neutral substances can be separated using surfactant micelles, instead of what happens in CE, which only separates ionic compounds. Briefly, the separation principle in MEKC is based on the addition of a surfactant, above its critical micellar concentration (CMC) to the buffer solution, acting like a micellar phase. This micellar phase will interact with the analytes present in the sample, by a partitioning mechanism, and then the analytes that have greater affinity for the micelles are trapped inside the micelle. The migration time of the analytes will depend on the affinity to the micelles. The analytes that have more affinity will have a slow velocity of migration, while the other analytes will migrate faster, and the retention time will be lower [51]. One of the first studies exploiting MEKC to separate AgNPs from wound dressing, for example, was developed in 2019 by M. Konop et al. [50]. The wound dressings used were two commercially available (Atrauman^®^ Ag (Sydney, NSW, Australia), Aquacel^®^ Ag (ConvaTec, Princeton, NJ, USA)) and one experimental (FKDP-AgNPs) dressing. Each sample was previously prepared before MECK analysis, by mixing at 300 rpm with fetal bovine serum (FBS) for a total of 72 h. Several aliquots were analyzed at different time intervals: 1, 24, 48, and 72 h. The study allowed to conclude that the best separation conditions for AgNPs were attained using 20 mM borate buffer solution at pH 9 and with 20 mM SDS addition. As a result, Atrauman Ag was the dressing where the quickest release of silver from the matrix was observed, whereas, in Aquacel Ag and FKDP-AgNPs, a slower silver release was observed. The observed results were very important, allowing to conclude that the dressing matrix influences the silver release. The proposed MEKC method required only a very small volume of sample extract (100 µL), and the execution of the procedure was fast and simple. However, due to undesired interactions between the synthetized AgNPs, used to produce in the lab a wound dressing, with the electrolyte solution, some instability of the NPs resulting in some aggregation phenomena was confirmed. In fact, the measured zeta potential value was about −42.8 ± 6.65 mV. Additionally, the method presented low reproducibility of the analytical signals on the electropherograms because AgNPs tended to sediment. The present work revealed that more studies of MEKC must be done to optimize the conditions of separation and quantification of AgNPs released from dressing materials, and other samples, involving more studies of interaction between the AgNPs and FBS used to extract the analytes from the samples.

### 2.6. Selective Precipitation

Polydisperse NPs can be separated into fractions through a size-selective precipitation technique, according to size-dependent physical and chemical properties, reactivity, and/or stability [13,52]. To achieve a selective precipitation of the NPs, by modifying their solubility in the medium, a miscible substance (gas, liquid, or salt) where the NPs do not aggregate is added into the suspension [11,52]. Following this addition and mixture, the nanoparticles gradually start precipitating, from the largest- to the smallest-sized, originating several fractions with size-separated NPs, which can be collected as they are formed. To attain this separation, there are four possible methods: (i) using a supercritical fluid that acts like a solvent with density-tunable dissolving power; (ii) adding a non-solvent where NPs do not solubilize; (iii) adding salts; and (iv) using a gas-expanded liquid [11,52]. Not all these approaches were found in the literature for separation of AgNPs, which was the aim of the present revision. Table 6 compiles the works properly identified in the literature containing a detailed description of selective size-selective precipitation of AgNPs. The analysis of the table leads to conclude that these separation processes are not much applied for the separation of AgNPs.

In 2005, McLeod et al. [53] developed a method to separate AgNPs using a CO_2_-expanded liquid approach. By adding a compressed gas (CO_2_) to an organic solvent (hexane), the liquid volume is expanded several times, which is why the resultant liquid CO_2_/hexane is described as a gas-expanded liquid or abbreviated by GEL. Briefly, the separation process was developed inside of a spiral glass tube. Here, 200 µL of a hexane solution containing the AgNPs were inserted, and the pressure was slowly elevated to 500 psi, which allowed to reach the equilibrium state over 20 min

When the GEL equilibrium was reached, the fraction with the largest AgNPs, which were no longer soluble in the expanded liquid, precipitated. Next, the spiral glass tube was rotated 180° to achieve other AgNPs populations that were attached in the tube walls. Increasing the gas pressure, a second fraction of AgNPs with small dimensions precipitated as well. The process continued until all AgNPs became precipitated. The gradual changes of gas pressure, from 500 psi to 650 psi, selectively precipitated AgNPs, from the largest to the smallest. In the end, six fractions of AgNPs were collected and analyzed by TEM. This technique allowed a precise size-separation of AgNPs in a single step, taking no longer than 1.5 h and requiring a reduced volume of organic solvent (200 µL); it was possible to select the nanoparticles to be separated, by size, by choosing the necessary pressure value. By testing a sample constituted by AgNPs of sizes ranging between 2 and 10 nm, with a mean size of 5.5 nm, the separation into six different mean diameter-sized fractions of AgNPs: 6.7 ± 1.4 nm, 6.6 ± 1.0 nm, 5.8 ± 1.1 nm, 5.3 ± 0.5 nm, 4.8 ± 0.5 nm, and 4.1 ± 0.6 nm was achieved. This new utilization of CO_2_-gas-expanded liquids provides the area of nanotechnology with a promising method for NPs purification and separation by size.

**Figure 7:**
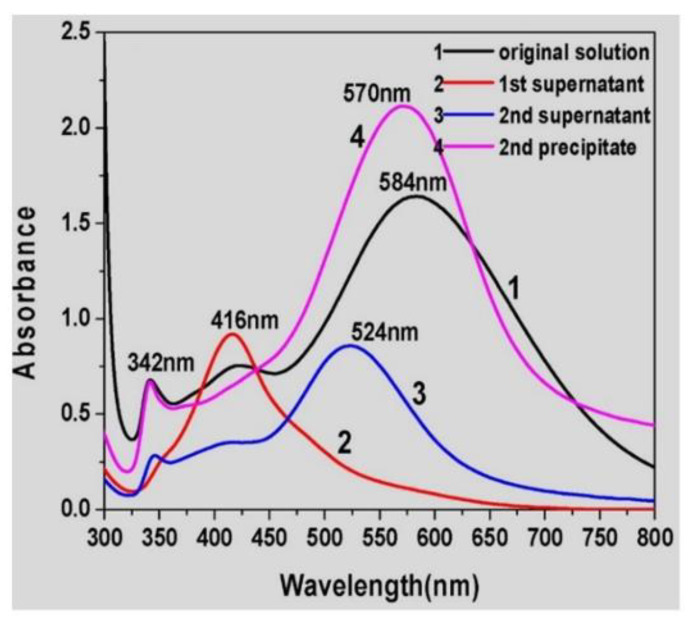
Separation of AgNPs by shape, monitored by UV–Vis spectrophotometry, after two sequences of selective precipitation. Reprinted from [54], copyright (2018), with permission from Elsevier.

To purify AgNPs of different shapes and sizes, prepared by a seed-mediated technique, in 2018, Hu et al. [54] applied a surfactant-assisted shape-separation method. The authors took a previously reported method used to purify AuNPs [55] and adapted it to purify AgNPs. The sample suspension was centrifuged at 10,000 rpm for 10 min, and then different volumes of different concentrations of CTAB solution (0.2–0.5 M) were added into the precipitate and mixed at different temperatures (25–80 °C). The authors studied the influence on the separation of AgNPs (triangular shape) of different CTAB volumes, CTAB concentrations, CTAB temperatures, and aging times. Following this, the study proceeded with secondary separations controlled by different CTAB concentrations and volumes. Finally, the authors tested the optimized procedure to separate silver nanotriangles and nanospheres. The bulk suspension was a mixture of silver nanotriangles, nanospheres, and nanorods. The initial UV–Vis spectrum related to the as-synthesized AgNPs presented three different absorption peaks (~342 nm, ~422 nm, and ~584 nm), while, after the first separation step, the supernatant showed a UV–Vis spectrum with a single absorbance peak at 416 nm, which is related with the presence of silver nanospheres with different sizes. After the second separation step, the supernatant had three absorption peaks (~342, ~416, and ~584 nm), which means that, in this phase, the AgNPs were mainly silver nanotriangles. With this approach, a successful separation was achieved, monitored by UV–Vis spectra (Figure 7). The relative ratio between CTAB and AgNPs seems to be determinant, together with the temperature, to separate the triangular-shaped AgNPs. Probably, the procedure requires the optimization of the separation conditions for each type of AgNPs under analysis. The authors concluded that this separation methodology could be used to separate not only AuNPs but also silver or other metal NPs.

### 2.7. Membrane Filtration

An alternative to the purification and size-separation of NPs is filtration through a porous membrane. The distribution of sizes of the membrane pores influences the time of retention and the elution of the NPs. For example, for nanoparticles with sizes ranging from 2–50 nm, an ultrafiltration process is used, whereas, for particles with a size ranging between 20–500 nm, the process is known as microfiltration [11,13]. In this method, the separation process is based on the migration of the particles through a porous membrane, because the concentration on one side is different from the other side. These concentrations’ differences make the nanoparticles diffuse from the higher-concentration to the lower-concentration area. The advantages of this method are the minimal equipment requirements and small solvent volumes, the recyclability of the feed solution, the high resolution, and the facility to be scaled up [11,56]. Nowadays, the most commonly used membranes are made of polymer or ceramic [56]. The size of the membrane pore is a key factor for an effective separation. So, if the membrane has a pore size smaller than the size of the nanoparticles to be purified, no separation will occur, because the NPs cannot simply cross through the pores by diffusion. Additionally, adsorption and aggregation of the nanoparticles can occur in these situations, causing the blockage of the membrane surface. [11]. The better uniformity of membrane’s porosity, the better the performance of nanoparticle separation [13].

To our knowledge, this technique was not used to separate AgNPs, but it was successfully used in the separation of AuNPs by Krieg et al. [56], in 2011. As with other separation techniques previously described, its use in noble-metal nanoparticles based on gold indicates the possible use in silver-based nanoparticles as well. Considering the recognized potentiality of the separation methods based on porous membranes, some discussion about related practical works found in the scientific literature was included here. In the work of Krieg et al., some supramolecular membranes were prepared from PP2b (5,5′-bis(1-ethynyl-7-polyethylene glycol-N,N′-bis(ethylpropyl) perylene-3,4,9,10-tetracarboxylic diimide)-2,2′-bipyridine) in water. To study the applicability of the fabricated membrane for ultrafiltration, AuNPs (red suspension) of various sizes were filtered over a 12-mm-thick PP2b layer. As a result, a pale-yellow filtrate was obtained, and characterization by UV–Vis spectrophotometry suggested that NPs larger than 5 nm were removed, by the absence of a surface plasmon band in the filtrate. To confirm these results, a TEM analysis was performed. The TEM images confirmed that NPs with a particle size > 5 nm were effectively removed and remained in the retentate, and, also, allowed to confirm that the membrane cut-off was 5 nm. To evaluate the membrane performance, the authors made an additional ten experiments, where five of them were performed with freshly prepared PP2b for membrane fabrication, and the other five were performed with the recycled membrane. As a result, and after TEM analysis, the authors obtained a particle size in the filtrates of about 2.3 ± 0.2 nm for the first five experiments (freshly membrane) and, for the recycled one, a particle size of about 2.3 ± 0.1 nm. These supramolecular membranes have the advantage of simple fabrication, versatility, and the capacity to be used multiple times, after being cleaned and recovered. The authors of the reviewed work concluded that these membranes allow to recover the separated nanoparticles, obtaining purified suspensions of AuNPs to further use in biological, analytical, and biochemical applications, which is an important feature to highlight, and that the method could be extrapolated to purify other nanoparticles with a cutoff of roughly 5 nm. Additionally, since the membranes are robust, and the supramolecular structure retains its adaptivity, they are susceptible to recycling.

### 2.8. Liquid Extraction

Extraction is a separation process where the analytes present on the sample (a liquid or a solid mixture) are selectively separated with a liquid immiscible solvent. Usually, the two immiscible phases are water and an organic solvent, and the separation is dependent on the relative solubility of the target analytes. Widely used, this is a method for organic and inorganic compounds [11,13].

**Figure 8:**
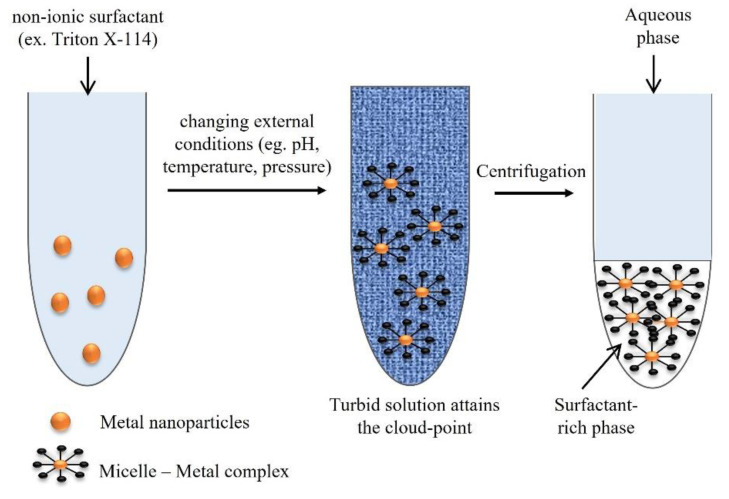
Representative scheme of CPE protocol.

In the scope of the extraction methods available, the concept of cloud-point refers to the minimum temperature in which a clear solution undergoes a liquid–liquid phase separation to form an emulsion. Thus, cloud-point extraction (CPE) is based on the solubilization ability and on the cloud points of non-ionic surfactants. It is considered an approach for the separation, extraction, and preconcentration of trace elements, before their determination [57], such as for beryllium and chromium in water samples [58], silver in environmental waters [59], cobalt in water and food samples [60], and cadmium, copper, and lead in biological fluids [61], among others. The extraction protocol for trace elements is based on three steps. In the first one, a non-ionic surfactant, for example, Triton X-114, is added into the sample solution with a concentration that exceeds the critical micelle concentration. Temperature, pressure, or pH can be changed to form micelles, in which trace elements are entrapped. At the cloud-point, the solution with the surfactant becomes turbid, and it is separated into two distinct phases (the surfactant-rich phase and a dilute aqueous phase). The analytes separated in the surfactant-rich phase can then be extracted and concentrated, due to the analyte–micelle interaction [1,57]. In the case of separation of AgNPs, the addition of some salts, such as NaNO_3_ or Na_2_S_2_O_3_, improves the separation of the phases and increases the efficiency of the extraction. In this situation, the separation is facilitated because the salt has the ability to reduce the Coulomb repulsion between charged AgNPs [62]. Additionally, the increase in the extraction’s efficiency occurs because S_2_O_3_^2−^ anion acts like a chelating reagent with silver, binding to the silver ions forming a complex, which prevents the transfer of Ag^+^ to the surfactant-rich phase, thus eliminating the Ag^+^ interference completely, allowing only the extraction of AgNPs. Nonetheless, S_2_O_3_^2−^ may cause some interference in the characterization of AgNPs by UV–Vis spectrophotometry. Thus, the authors recommend the use of NaNO_3_ when the objective is a UV–Vis characterization, but in the case of other analyses, the use of Na_2_S_2_O_3_ is preferable [59]. Figure 8 shows the schematic representation of the CPE protocol steps.

CPE is also a method of choice for extracting pollutants (e.g., metal NPs) from environmental and biological samples, without modifying their sizes and shapes. It is a simple, low-cost, and environment-friendly technique, which has a high capacity to concentrate trace elements [57]. Nevertheless, a limitation of this method is the low extraction efficacy when the analytes are protein-coated NPs, like AgNPs functionalized with BSA. As an alternative, Qu et al. [48] proposed a capillary electrophoresis approach for separating those AgNPs, allowing to achieve excellent recovery values in consumer products tested (higher than 93%).

Table 7 contains the analysis of some works found in the scientific literature, involving the separation of AgNPs based on extraction procedures.

**Figure 9:**
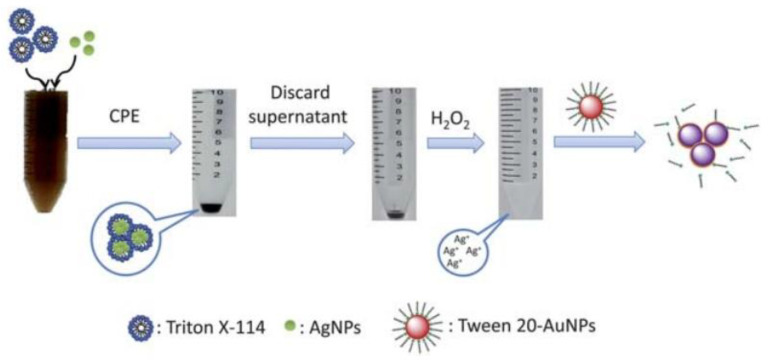
Separation of AgNPs by Triton X-114-based CPE. Reproduced from Ref. [63] with permission from the Royal Society of Chemistry.

From the analysis of the Table 7, it is evident the prevalence of CPE techniques to separate the AgNPs. For example, in the work of Wu et al. [63] in 2011, a methodology to separate AgNPs and Ag^+^ in environmental waters was developed, while at the same time, the quantification of AgNPs was developed. For this purpose, the authors combined CPE and a colorimetric assay using Tween 20-stabilized AuNPs to allow the indirect quantification of AgNPs. The extracting agent Triton-X 114 was used in the preconcentration step of CPE, and Tween 20-stabilized AuNPs were used as a colorimetric probe, for Ag^+^ obtained after, by oxidation with H_2_O_2_ of the separated fraction of AgNPs. Figure 9 illustrates the procedure used by the authors for the identification of AgNPs through the AuNPs-based colorimetric probe. For the detection of Ag^+^ through colorimetric assay after CPE, the supernatant was discarded, and an amount of H_2_O_2_ was added to the concentrated AgNPs present in the Triton-X-114-rich phase. H_2_O_2_-oxidized AgNPs were added to Ag^+^ under acidic conditions. The Tween 20-AuNPs act as sensing probes to the silver ions. The presence of the remains of citrate ions on the AuNPs surface reduces Ag^+^ to Ag on the surface of AuNPs, and, as consequence, the stabilizer Tween 20 is removed from the AuNPs. As a result, the AuNPs became unstable and aggregate. The developed method proposed by the authors showed high selectivity for AgNPs and allowed to separate the AgNPs from Ag^+^.

With the developed work, the authors were able to propose for the first time a new methodology, combining CPE with AuNPs-based sensors, to separate and detect AgNPs. Furthermore, even in the presence of low AgNPs concentrations in the samples, this approach can be used without requiring equipment, since the changes in color of the AuNPs can be observed with the naked eye. So, in the future, this methodology can hopefully be widely applied to the in situ AgNPs screening in environmental waters. This potentiality of the method is worthy of much consideration for its importance in the real-time control of AgNPs pollution in the environment.

**Table 7:** Overview of the AgNPs separation by liquid extraction procedures.

**Separation Method**	**Detection Method**	**Analyte**	Size (nm)	Matrix	Recovery (%)	LOD Value	Optimal Separation Conditions	Year
Selective extraction with *n* alkanoic acids (*n*-decanoic acid) [64]	-	Au and Ag dendrimer encapsulated NPs (DENs)	HR-TEM: 1.4 ± 0.4 for G6-OH (Au_147_) and 1.7 ± 0.4 for G6-OH (Ag_110_)	Aqueous medium	-	-	4.0 mL of 0.25 M *n*-decanoic acid/hexane solution; 6.0 mL of an aqueous mixture containing 0.18 mM G6-OH(Ag110) and 0.20 mM G6-OH(Au147); vortex for 30 s	2004
Triton X-114-based CPE [65]	-	Au NPs, Ag NPs, C60 fullerene, TiO_2_, Fe_3_O_4_ NPs, CdSe/ZnS, and SWCNTs	TEM: 5–100 nm	Aqueous medium	92–97%	-	Triton X-114 surfactant (3.6 mM); NaCl (3.4 mM); heat the suspension above the CPT (23–25 °C)	2009
Triton X-114-based CPE [63]	Spectrophotometry	AgNPs	TEM: 10 and 54 nm	Environmental water	Tap water: 102 ± 3%; seawater: 98 ± 5%	Tap water: 4.3 ng/mL; seawater: 43 ng/mL	0.01 M Na_2_S_2_O_3_; 0.2% Triton X-114; incubate at 40 °C, 30 min; centrifuge at 750× *g* at room temperature, 5 min	2011
Triton X-114-based CPE [59]	ICP-MS	AgNPs	TEM and SEM: 9–94 nm	Environmental water	57–116%	0.006 µg/L	1 M Na_2_S_2_O_3_ or 3.5 M NaNO_3_; 5% (*w/v*) TX-114; pH 3; incubate at 40 °C, 30 min; centrifuge at 2000 rpm, at room temperature, 5 min	2009
Triton X-114-based CPE [62]	ICP-MS	AgNPs	TEM: 12.4 ± 0.3 nm	HepG2 Cells	Approx. 92%.	2.94 μg/L for AgNPs and 2.40 μg/L for Ag^+^	1 mol/L Na_2_S_2_O_3_; 10% (*w*/*v*) TX-114; pH 3,5; incubate at 40 °C, 30 min; centrifuge at 3000 rpm, at room temperature, 5 min	2013
Triton X-114-based CPE [66]	ICP-MS	AgNPs	-	Environmental waters and antibacterial products	1.2–10% for Ag^+^ and 71.7–103% for AgNPs	0.4 μg/kg for AgNPs and 0.2 μg/kg for Ag^+^	1 M Na_2_S_2_O_3_, and 10% (*w/v*) TX-114; pH 3; incubate at 40 °C, 30 min; centrifuge at 2000 rpm, at room temperature, 5 min	2011
Triton X-114-based CPE [67]	ETAAS	AgNPs	-	Environmental water	>88%	0.7 ng/L	1.0 mL of saturated EDTA solution; 400 mL of 1 M sodium acetate; 100 mL 1.25 M acetic acid; 1 mL of 10% (*w/w*) TX-114; incubate at 40 °C, 2 h	2013
Triton X-114-based CPE [68]	ETAAS	AgNPs	20, 40, and 60 nm	Waste water	110 ± 6% and 101 ± 10%	0.04 µg/L	8.6% (*v/v*) Triton X-114; saturated EDTA; pH 7; incubate 60 °C for 20 min; Centrifuge at 7000 rpm, at 4 °C, 20 min	2018
CPE [69]	TXRF	AgNPs	SEM: 40–100 nm	Soil extracts and consumer products water extracts	-	0.7–0.8 µg/L	1 M Na_2_S_2_O_3_; 5% Triton X-114; pH 3,7; incubate at 40 °C, 30 min; centrifuge at 2000 rpm, at room temperature, 5 min; cool in a freezer for 15 min	2018

CPE: cloud-point extraction; ICP-MS: inductive coupled plasma mass spectrometry: ETAAS: electrothermal atomic absorption spectrometry; TXRF: total reflection X-ray fluorescence spectrometry; TEM: transmission electronic microscopy; SEM: scanning electronic microscopy.

In 2009, Liu et al. [59] developed a CPE method with Triton-X 114 to preconcentrate AgNPs from environmental water, without disturbing their sizes and shapes. The AgNPs were concentrated into the Triton-X 114-rich phase, and then they were characterized by TEM, SEM, and UV–Vis and quantified by ICP-MS after microwave digestion. Furthermore, the authors studied the optimization of the CPE parameters, studying, for example, the addition of some salts, like NaNO_3_ and Na_2_S_2_O_3_, and they proved that the addition improved the phase separation and the extraction of AgNPs, as well as the preservation of Ag^+^ in the upper aqueous phase, as previously explained in the introduction to the CPE concept. To evaluate the applicability of the optimized method (the optimized conditions can be consulted in the Table 7), the authors analyzed four types of real environmental water samples (influents, effluents, lake water, and river water). As a result, recoveries of 57–116% and a LOD of 0.006 µg/L were obtained. In conclusion, the proposed methodology showed to be efficient for the selective extraction of AgNPs and concentrations of trace AgNPs from environmental-water samples. Taking into account the previously described method in 2009 [59], in 2011, Chao et al. [66] had as the main goal the study of the applicability of the Triton-X-114-based CPE for the speciation analysis of AgNPs and Ag^+^ in environmental waters and antibacterial products. Six antibacterial products were tested, but AgNPs were detected in only three of them. The authors justified the results with the hypothesis that the actual values of AgNPs might be below the method’s LOD. Extraction efficiencies of Ag^+^ ranging from 1.2–10% were obtained, while the recoveries of AgNPs were between 71.7–103%, in antibacterial products, and 74.5–108% in environmental waters. The recoveries obtained for water samples were in agreement with those obtained in the previous study. The authors described this technique as fast (the maximum of the extraction efficiency was reached in 10 min), simple, and low-cost.

In 2013, Yu et al. [62] proposed to separate AgNPs and Ag^+^ in the cell lysates of exposed HepG2 cells using Triton-X-114-based CPE. After the exposure of HepG2 cells to AgNPs, the cells were centrifuged to cause cell lysis, and cell lysates were obtained. Then, Na_2_S_2_O_3_ was added to the cell lysates, to be subjected to CPE. As already mentioned, the addition of the salt allowed the preservation of Ag^+^ in the upper aqueous phase and facilitated the transference of AgNPs into the Triton-X-114-rich phase. The AgNPs and Ag^+^ contents were determined by ICP-MS, after microwave digestion. The LOD of this method was calculated as 2.94 μg/L for AgNPs and 2.40 μg/L for Ag^+^. The results showed an average total recovery of ≈92%. The proposed methodology allowed the quantification of AgNPs and Ag^+^ contents in a complex matrix, like cell lysates. For the first time, a method that allowed this determination in biological samples was developed.

A quantification procedure for AgNPs based on electrothermal atomic absorption spectrometry (ETAAS) was developed by Hartmann et al. [67] (2013). The authors had to first extract the analytes through a CPE method, from environmental samples. The authors re-evaluated the optimized conditions for the AgNPs separation by CPE, since, in the proposed work, the detection system was based on ETAAS. Following this, it was concluded that a pH value ≥ 5 and ethylenediamine tetraacetic acid (EDTA) as the complex agent were better conditions for the CPE enrichment factor. In Table 7, one can find the optimized conditions for the assay, including the ETAAS measurement parameters. Under the optimized conditions, a LOD of 0.7 ng/L was obtained, and the environmental analysis of the samples (river water and treated and untreated municipal wastewater) revealed a mean recovery > 88% in all cases. The described methodology was fast; allowed for an easy sample preparation, by avoiding the microwave digestion before the quantification by ETAAS; and allowed to achieve a low LOD value. Despite the encouraging results, the authors concluded that more studies must be done to evaluate this separation technique, such as using AgNPs of different syntheses with several stabilizing agents. In fact, the authors had to re-evaluate the CPE conditions for the application of the extraction method coupled to ETAAS.

More recently, in 2018, Lopez-Mayan et al. [68] developed a reliable and simple CPE method to purify AgNPs from wastewater samples from three water-treatment plants from Galicia, Spain, followed by quantification by ETAAS. Through multivariate analysis (and experimental-design approaches), the parameters related to the CPE method (surfactant concentration, type of complexing agent (EDTA or Na_2_S_2_O_3_), pH value, incubation temperature, incubation time, and centrifugation time) were optimized. The selected optimal conditions were 8.6% (*v/v*) Triton X-114, 750 µL saturated EDTA, and pH 7. To evaluate the sensitivity of the procedure, the LOD and the limit of quantification (LOQ) were calculated as 0.04 µg/L and 0.13 µg/L, respectively. The recovery assays revealed results of 110 ± 6% and 101 ± 10% for the sample spiked with 1.0 and 2.0 µg/L of AgNPs, respectively. The authors concluded that the combination of CPE and ETAAS was a good approach to analyze AgNPs in wastewater samples and that it was a valid alternative to CPE coupled to ICP-MS.

The combination of the CPE method with total reflection X-ray fluorescence spectrometry (TXRF) was made for the first time, in 2018, by Torrent et al. [69]. TXRF allows materials characterization and trace-element determination based on the principle of total reflection of X-rays. Briefly, when an incident X-ray beam, with an angle below the critical angle of the substrate, falls upon a flat polished surface, a fluorescence signal characteristic of the present element is emitted from the sample [70]. This combination procedure was used to separate and quantify AgNPs, with different coatings (citrate, PVP, and PEG), in complex aqueous samples, including soil and consumer-product water extracts. In all cases, the LOD obtained was 0.7–0.8 µg/L. The authors also studied the influence of the presence of Ag ions in the extraction and recovery of AgNPs, by the developed CPE-TXRF methodology. It was concluded that for Ag^+^/AgNPs ratios higher than three, the procedure was not adequate since it was verified that, in those conditions, the separation between Ag^+^ and AgNPs was not efficient. Yet, considering the usual ratios present in most real samples, this was not an issue. Following this, the CPE-TXRF methodology was applied for the quantification of AgNPs in the samples of Hansaplast Universal Antibacterial Plasters (Beiersdorf AG, Hamburg, Germany) and spiked soils. The samples were submitted to an aqueous extraction procedure, followed by filtration using an acetate cellulose membrane filter of 0.45 µm pore size. The CPE procedure followed by the authors was based on the work of Liu et al. [59], with the proper adaptions derived from optimizations studied in the work. The obtained results by the proposed methodology were confirmed by single-particle inductively coupled plasma mass spectrometry (SP-ICP-MS) analysis.

A good agreement was obtained by comparing both analytical techniques, CPE-TXRF and SP-ICP-MS, with respect to AgNPs quantifications in two soil samples spiked with AgNPs. In relation to the extract sample from the Hansaplast band aid, the estimated concentrations were 60 ± 5 µg/L with CPE-TXRF and 91% of AgNPs by SP-ICP-MS. In this case, the concentrations estimated by CPE-TXRF were much lower than the actual total Ag concentration (76% lower comparing with the obtained result by ICP-OES, which estimated the total silver content). Since the SI-ICP-MS technique originated results that indicated the presence of a large distribution in the sizes population of AgNPs, it could be that the CPE extraction efficiency was affected by the presence of larger AgNPs, which is also supported by some reports found in the literature [71] regarding this same problem. The authors concluded that more studies must be performed to improve the combination techniques for screening AgNPs in mixtures with different nanoparticles, namely, of different coatings and particle sizes.

## 3. Timeline Conceptual Evolution

A scientific research at the global citation database Web of Science^TM^ was conducted, using an advanced search for each separation method analyzed in this study. By restricting the research to silver nanoparticles and the separation method, this survey initially revealed the following number of studies for each method: 318 for the magnetic-based method, 49 for hydrodynamic forces, 203 for chromatography, 55 for centrifugation, 99 for electrophoresis, 14 for selective precipitation, 72 for membrane filtration, and 120 for liquid extraction. Yet, after consulting the studies, it was found that most of them were not in fact focusing on silver nanoparticles as the target of separation from crude suspensions or other samples. Additionally, some studies were also excluded due to other aspects, namely, the inclusion of AgNPs in the separating materials (like in magnetic nanocomposites or as antiseptic additives in filtration membranes) or their use as probes. Consequently, after a thorough and refined analysis of the previous studies, the numbers of the survey were reduced to: 5 for the magnetic-based method, 21 for hydrodynamic forces, 16 for chromatography, 11 for density gradient centrifugation, 22 for electrophoresis, 2 for selective precipitation, 0 for membrane filtration, and 20 for liquid extraction.

Using the numbers mentioned before, a timeline conceptual evolution study was conducted to allow the perception of any possible tendency in the use of a specific separation method and if there is evidence of more investment in research to a separation procedure, during the time between 2004 and 2020.

**Figure 10:**
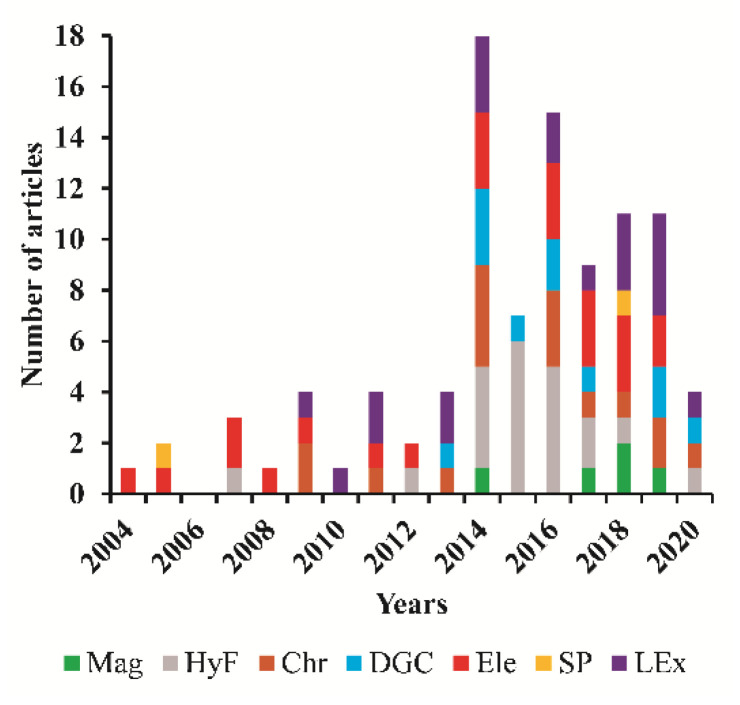
Timeline of separation methods: graphic representation of the number of published articles discriminated by separation method (separation methods: Mag—magnetic-based schemes; HyF—hydrodynamic forces; Chr—chromatography; DGC—density gradient centrifugation; Ele—electrophoresis; SP—selective precipitation; Lex—liquid extraction).

A graphic representation of the distribution of the reviewed works by years, accounting for the number of each separation technique, is depicted in Figure 10. A relatively higher number of published studies in the years 2014 and 2016 was identified, whilst no studies in 2006 were identified. As already stated, the most-exploited methods of separation of silver nanoparticles identified for analytical purposes were electrophoresis, hydrodynamic, and liquid-extraction-based methods.

Unfortunately, the identified number of articles found in the literature in the scope of the present review was low, revealing that if one considers the large number of studies per month or year that are published about silver nanoparticles, most of them do not detail or reveal any separation or purification methods for the nanoparticles. In our opinion, this should change, because the influence of the surrounding medium resultant from the synthesis procedures contains several remains of compounds that influence the assays where the AgNPs are intervenient. This is important because it leads to biased conclusions about the real influence or effect of silver nanoparticles in, for example, cellular assays, electronic applications, drug-delivery effects, decontamination assays of the environment, etc. For example, in cellular assays, it is of utmost importance to purify the nanoparticles to be tested with cells, to achieve true and real conclusions about the nanoparticles’ effects, reflected, for example, in calculated IC50 values. In the work of Morais et al. (2020) [72], the authors discussed the problem of hundreds of works dealing with AgNPs synthesized by green chemistry, with natural products (as plant extracts) in which the authors did not purify or separate the nanoparticles but nonetheless conducted cell assays with cancer-cell lines, calculating IC50 values. However, in the literature, one can also find works using the same plant extracts tested in tumor cells, with the respective IC50 values also determined. So, there is no way to be sure if the IC50 values were only due to the action of the AgNPs. This doubt has a significant impact on the nanotechnology-based health-treatment evolution. Fahad A. Alharbi et al. (2020) [73] synthesized AgNPs using the leaves extract of a xerophytic plant (*Neurada procumbens*). After the synthesis, the authors tested the antibacterial properties of the produced AgNPs against a group of multi-drugs-resistant (MDR) bacteria (*Klebsiella pneumoniae, Acinetobacter baumannii*, and *Escherichia coli*). As a result, the AgNPs exhibited a high level of antimicrobial activity against MDR bacteria. However, after synthesis, the suspension of AgNPs did not undergo any purification process, so, probably the effects observed against the MDR bacteria might in fact be due to the action of the silver nanoparticles capped with the leaves-extract compounds, but they could also be due to the residues of the natural product that resulted from the entire synthesis process.

So, one can conclude that more research should be performed focusing on the theme of this review since there is a noticeable lack of innovation or application of separation methods in original works involving silver nanoparticles and their use in a purified format.

## 4. Conclusions

The separation/purification process of AgNPs is of utmost importance since the applicability of the NPs depend on its purity. In all possible applications for AgNPs, one wants to take advantage only of the physical–chemical properties of the NPs and of from the precursors and reagents remaining from the syntheses. A variety of analytical procedures have been developed to separate AgNPs from the crude synthesis material or other matrices, allowing its purification and size sorting. In this review, we thoroughly described the current options for the separation/purification methods of NPs, mainly the silver ones from diverse sample matrices.

Over the last few years, significant progress has been made, derived from technology advances. We reviewed successful cases of separation of AgNPs. Despite considering the advantages and disadvantages of these processes, it is not possible to conclude which, among all, is the best method to apply. The range of existing separation and purification methods is diverse, and the most-suitable method will depend on the type of NPs to be separated and on the purpose that will be given to them. Depending on that purpose, it might be imperative to maintain the integrity of the AgNPs for further applications. In these cases, one must be aware that some separation methods end up disintegrating the AgNPs making unfeasible their further use, restricting the choice of the method. However, traditional methods still must undergo some optimizations to be more efficient and to follow environmental sustainability demands. There is a greater need to develop novel methods or to innovate others already implemented, allowing a more-efficient AgNPs separation, while, at the same time, ensuring a low-cost, versatile, and eco-friendly method.

## Figures and Tables

**Table 1:** Overview of AgNPs separation based on magnetic schemes.

Separation Method (Ref.)	Size (nm)	Matrix	Recovery (%)	Optimal Separation Conditions	Year
Surface-modified magnetic capture particles (UMP, GMP, DMP, and Mix D–G) [15]	SEM: 10 and 75	Environmental water	>99%	Add 2 μg/mL AgNPs suspension to 2 mg/mL magnetic particles; shake the mixture for 30 min at 100 rpm to disperse the particles; incubate for 15 min to facilitate absorption of the AgNPs	2014
Magnetic reduced graphene oxide [16]	TEM: 30, 50, 80, and 100 (citrate); 60 and 100 (PVP)	Environmental water	98%	Add 10 mg of adsorbent to 10 mL of the AgNP/Ag suspension; at RT (25 ± 0.5 °C), oscillate at 200 rpm	2017

UMP: unmodified magnetic particles; GMP: glutathione-functionalized magnetic particles; DMP: dopamine-functionalized magnetic particles; Mix D–G: equal mass mixture of DMP and GMP; TEM: transmission electronic microscopy; SEM: scanning electronic microscopy; RT: room temperature.

**Table 2:** Overview of AgNPs separation based on hydrodynamic forces.

Separation Method (Ref.)	Size (nm)	Matrix	Recovery (%)	Optimal Separation Conditions	Year
SdFFF [24]	FE-SEM: 20–100 and 60–150	Environmental water	-	Carrier liquid: water with 0.1% FL-70; injection volume 5~30 µL; vortex for 30 s before the injection; RT	2007
Flow FFF [25]	TEM: 15	-	-	Channel flow: 1 mL/min; cross flow: 0.4–1 mL/min	2009
AF4 coupled with ICP-MS [26]	TEM: 10 nm, 20 ± 5, 40 ± 5, 60 ± 5, and 80 ± 7 nm	Two consumer products, an antiseptic, and a dietary supplement	83 ± 8% and 93 ± 4%	Ultrafiltration membranes, cut-off 1 and 4 kDa; flow rate of 0.8 mL/min; mobile phase: 0.01% SDS, at pH 8	2011
AF4 coupled with ICP-MS [27]	TEM: 42 ± 10 nm	Aqueous medium	<1%	Carrier liquid: 0.5 mM NH_4_HCO_3_, pH 7.4; PES membrane, cut-off 10 kDa; flow rate: 1.0 mL/min; injection volume: 0.2 mL/min	2013
AF4 coupled with ICP-MS [28]	TEM: 10, 40, and 60 nm	Commercial nutraceutical Products and Korean beer	97 ± 2 and 106 ± 1%	Carrier liquid: ultrapure water and SDS 0.01% at pH 8; regenerated cellulose Membrane, cut-off 10 kDa; injection volume: 200 µL	2014
HF5 coupled with ICP-MS [22]	TEM: (tannic acid) 11.4, 5.9, 9.1, 26.5, 8.9; (citrate) 10 and 15.5	-	Similar for both capping agents	Carrier liquid: 30 mM TRIS buffer (pH 8) and 0.02% (*w/v*)—FL-70 and NaN_3_ (pH 10); flow rate: pump A—0.5 mL/min, pump B—2.0 mL/min.	2015
HF5 coupled with MALS [23]	TEM: 20 and 140 nm	Aqueous media	>90%	Polymeric membrane, cut-off 100 kDa; mobile phase: water; injection volume: 4 µL	2015
HF5 coupled with multiple detectors (UV-Vis, DLS, and ICP-MS) [29]	TEM: 1.4, 10, 20, 40, and 60 nm	Lake and river waters	70.7−108%	Carrier liquid: 0.1% (*v/v*) FL-70 with 0.02% (*w/v*) NaN_3_; inlet flow rate, 1.50 mL/min; radial flow rate, 0.70 mL/min; axial flow, 0.80 mL/min; focusing time: 4 min	2015

FFF: field-flow fractionation; SdFFF: sedimentation field-flow fractionation; AF4: asymmetrical flow field-flow fractionation; HF5: hollow fiber field-flow fractionation; MALS: multi-angle light scattering; ICP-MS: inductive coupled plasma mass spectrometry; DLS: dynamic light scattering; UV–Vis: ultraviolet–visible spectrometry; FL-70TM: commercially available mixture of nonionic and anionic surfactants that included oleic acid, sodium carbonate, tergitol, tetrasodium EDTA, polyethylene glycol, and triethanolamine; TRIS buffer: 2-amino-2-hydroxymethyl-propane-1,3-diol; TEM: transmission electronic microscopy; SEM: scanning electronic microscopy; FE-SEM: field emission scanning electron microscopy; RT: room temperature.

**Table 3:** Overview of AgNPs separation based on chromatography.

Separation Method	Size (nm)	Matrix	Recovery (%)	LOD Value	Optimal Separation Conditions	Year
Reversed-HPLC coupled with ICP-MS [31]	TEM: 10, 20, and 40 nm	Fetal bovine serum and textile products	>80%	0.08 and 0.4 ng/L	Column: Nucleosil, 7 μm particle size, C18, 1000 Å pore size, 250 mm × 4.6 mm; flow rate: 0.5 mL/min; injection volume: 10 μL; mobile phase: 10 mmol/L ammonium acetate at pH 6.8 and 10 mmol/L SDS	2013
Hydrodynamic chromatography coupled with ICP-MS [32]	TEM: <100	Sewage sludge supernatant	-	2.3 ng/mL	Mobile phase: 0.002 M Na_2_HPO_4_; 0.2% non-ionic surfactant; 0.05% SDS; 0.2% formaldehyde; pH~7.5; injection volume: 20 µL; flow rate: 1.7 mL/min	2009
Reversed-HPLC coupled with ICP-MS in combination with isotope dilution analysis [33]	20, 30, and 40 nm	-	-	1000 Å column: 0.09–3.73 µg/L	Column: Nucleosil, 7 µm particle size, C18, 1000 Å pore size, 250 mm × 4.6 mm; mobile phase: 10 mmol/L SDS, 10 mmol/L ammonium acetate, penicillamine at pH 6.7; flow rate: 0.5 mL/min	2016
SEC coupled with ICP-MS [34]	TEM: 10, 20, 40, 60, and 100 nm	Antibacterial products and environmental waters	84.7–96.4% for Ag(I) and 81.3−106.3% for NAg	0.019 μg/L	Column: 500 Å pore-size; mobile phase: water containing 0.1% (*v/v*) FL-70 and 2 mM Na_2_S_2_O_3_; flow rate: of 0.7 mL/min	2014
SEC coupled with ICP-MS [35]	HR-TEM: 10, 20, and 30 nm	Biological tissues (rat liver)	73.7–113% in swine liver; 84.0–104% in rat liver	0.1 μg/g	Column: 5 μm particle size, 1000 Å pore size, 4.6 mm × 250 mm; mobile phase: 2% (*v/v*) FL-70 and 2 mmol/L sodium thiosulfate; flow rate: 0.5 mL/min	2018
Counter-current chromatography [36]	SEM: 13.7 ± 1.9, 14.1 ± 3.5, 19.2 ± 4.3, and 22.2 ± 4.9 nm	Phosphate buffer (20 mM, pH 11)	-	-	Mobile phase: hexane/toluene (1:1, *v/v*), 0.02 mM TOAB; injection volume: 5 mL, flow rate: 1 mL/min; oven temperature 20 °C; rotation of the chromatograph: 700 rpm	2009

ICP-MS: inductive coupled plasma mass spectrometry: HPLC: high-performance liquid chromatography; SEC: size exclusion chromatography; FL-70TM: commercially available mixture of nonionic and anionic surfactants that included oleic acid, sodium carbonate, tergitol, tetrasodium EDTA, polyethylene glycol, and triethanolamine; TEM: transmission electronic microscopy; HR-TEM: high-resolution transmission electronic microscopy; SEM: scanning electronic microscopy; FE-SEM: field emission scanning electron microscopy.

**Table 4:** Overview of AgNPs separation based on centrifugation.

Separation Method	Size [nm]	Shape	Matrix	Optimal Separation Conditions	Year
Sucrose density gradient centrifugation method [42]	FE-SEM and TEM: 15–235 nm	-	Chitosan-coated AgNPs	10%, 20%, 30%, and 40% sucrose gradient, during 2 h at 6000 rpm	2019
Sucrose density gradient centrifugation method [43]	HR-TEM: 52–117 nm for AuNPs, and from 38–61 nm for AgNPs	Spherical, pentagonal, triangular, and hexagonal	*Magnolia kobus* leaf extract AgNPs	40 min at 3500 rpm for AuNPs, and 90 min at 3500 rpm for AgNPs	2014
Centrifuging process [44]	-	Quasi-spherical	PVP-coated AgNPs	1st centrifugation: 8000 rpm; 2nd centrifugation: 16,000 rpm; 3rd centrifugation: 24,000 rpm	2015

HR-TEM: high-resolution transmission electronic microscopy; FE-SEM: field emission scanning electronic microscopy.

**Table 5:** Overview of the AgNPs separation by electrophoresis.

Separation Method	Analyte	Size [nm]	Matrix	Optimal Separation Conditions	Year
Agarose gel electrophoresis [46]	AgNPs with PEG	-	-	0.2% agarose gel; 30 min at 150 V; 0.5× TBE buffer (pH ≈ 9)	2007
CE with diode-array detection [47]	AgNPs	SEM: 36.3 ± 5.9 nm	-	Background electrolyte: 20 mM SDS,10 mM Tris, pH 8.5; voltage: 20 kV	2004
CE coupled with ICP-MS [48]	AgNPs with citrate acid, lipoic acid, PVP, and bovine serum albumin	TEM: 10–110 nm	Consumer products (six dietary supplements)	Background electrolyte: CHES 10 mM, TX-100 30 mM, pH 9.5; voltage: 25 kV	2015
CE [49]	AgNPs with honey or glucose	TEM: 12 and 18 nm	-	Background electrolyte: 20 mM sodium borate, 20 mM SDS, pH 8.5; voltage: 20 kV	2017
MEKC [50]	Wound dressings: Atrauman^®^ Ag, Aquacel^®^ Ag, and FKDP-AgNPs	PSD: 284.5 nm	Wound dressings	Background electrolyte: 0.02 M borate buffer solution, 0.03 M SDS; pH 9; voltage: 20 kV	2019

ICP-MS: inductive coupled plasma mass spectrometry; PSD: particle-size distribution; CE: capillary electrophoresis; MEKC: micellar electrokinetic chromatography; TEM: transmission electronic microscopy; SEM: scanning electronic microscopy; PVP: polyvinylpyrrolidone; PEG: polyethylene glycol.

**Table 6:** Overview of the AgNPs separation by selective precipitation.

Separation Method	Size (nm)	Optimal Separation Conditions	Year
CO_2_-expanded liquid approach [53]	TEM: 2 and 10 nm, having a mean size of 5.5 nm	Pressurization series: 500, 550, 600, 625, and 650 psi	2005
Surfactant-assisted shape [54]	-	1st separation: 0.4 mL of 0.4–0.5 M CTAB; 40–80 °C; 12 h aged 2nd separation: centrifugation at 400× *g*, 16 min; 0.2 mL of 0.2 M CTAB	2018

TEM: transmission electronic microscopy.

## Data Availability

Not applicable.
